# Revisit of the admitted control RND efflux pumps’ inhibitor PAβN: use limitations, and structural revelation in complex with MexB

**DOI:** 10.3389/fphar.2026.1906963

**Published:** 2026-10-19

**Authors:** Hilal Wehbi, Elodie Olivier, Quentin Cece, Maxime Mégret, Mélanie Sébastien, Nesrine Aissaoui, Catherine Llanes, Gilles Phan, Isabelle Broutin

**Affiliations:** 1 Université Paris Cité, CNRS, CiTCoM, Paris, France; 2 Université Marie et Louis Pasteur, Chrono-environnement UMR CNRS 6249, Besançon, France

**Keywords:** antibiotic resistance, efflux pump inhibitor (EPI), MexB transporter, phenylalanine-arginine beta-naphthylamide (PaβN), *Pseudomonas aeruginosa*, RND efflux pump, structure, toxicity

## Abstract

**Introduction:**

To bypass the health problem of antibiotic resistance in Gram-negative bacteria, a tempting approach is to use efflux pump inhibitors (EPI) to block the transporters before they expel antibiotics from the cell. One well-known EPI is the phenylalanine-arginine beta-naphthylamide (PAβN). Although it is described as toxic and therefore unsuitable for clinical use, it is highly effective and is commonly used as EPI reference compound in drug development. Nevertheless, the lack of toxicity on several cellular strains in our hands required us to revisit this known RND inhibitor, to redefine its limits.

**Methods:**

The rational design of new EPIs requires the integration of complementary information on their microbiological activity, toxicity toward eukaryotic cells, and description of molecular interaction with their target at atomic resolution. These parameters should also be considered in comparison with those of an established reference compound such as PAβN. Accordingly, the effect of PAβN on *Pseudomonas aeruginosa* was evaluated by determining the minimum inhibitory concentrations of three β-lactam antibiotics in the presence or absence of PAβN and by defining concentrations that do not perturb the bacterial envelope. Its toxicity was assessed using four human cell lines and multiple cellular endpoints. Finally, the interaction of PAβN with the RND transporter MexB was characterized by cryo-electron microscopy and crystallography.

**Results and Discussion:**

Microbiological analyses defined concentration ranges in which PAβN can be used to investigate efflux inhibition without causing detectable perturbation of the bacterial envelope. Across the four human cell models, PAβN displayed limited cytotoxicity, whereas β-naphthylamine was markedly more toxic. This points to the β-naphthylamide moiety as a potential contributor to PAβN toxicity, possibly resulting from metabolic proteolysis processing. Structural analyses revealed PAβN bound to MexB in the Tight conformation in both cryo-EM and crystal structures, but at distinct positions within the binding cavity. Comparison with the previously reported structure of PAβN bound to the artificially symmetric trimer of AcrB provides new insights into PAβN recognition and handling by RND pumps. Together, these microbiological, toxicological, and structural data refine the use of PAβN as a reference EPI and provide new perspectives on substrate transport by RND efflux pumps.

## 1 Introduction

In the context of bacterial antibiotic resistance, there is an urgent need to develop new drugs to counter the increasing emergence of resistant bacteria, especially concerning the Gram-negative bacteria for which very few new drugs have been validated to be used in clinic since more than 20 years. Among the mechanisms developed by Gram-negative bacteria to resist antibiotics’ treatments is the active efflux of toxic compounds out of the cell *via* tripartite RND efflux pumps (Darby et al., 2023; Wilhelm and Pos, 2024), preventing drugs from reaching their targets. One strategy to counter these weapons is to search for inhibitors that can block the efflux pump transporter localized in the inner membrane. In the present study, we focused our interest on MexB*,* the transporter of the constitutively expressed RND pump MexAB-OprM from *Pseudomonas aeruginosa*. MexAB-OprM is constituted of three proteins having different roles in the mechanism of transport (Tsutsumi et al., 2019; Glavier et al., 2020). The first step of the efflux mechanism is performed by MexB, an asymmetric trimer that uses the proton motive force as an energy source to efflux molecules. Each monomer can adopt a different conformation called LTO for Loose, Tight, Open, to push up the substrates through tunnels and cavities to expulse them in the pipe formed by MexA. Even if several combinations of the L, T, and O conformations have been observed (Müller and Pos, 2015), most of the solved structures of RND transporters adopt a LTO conformation, especially when in complex with substrates or inhibitors, that are mainly found in the T monomer structure (Alav et al., 2021). To develop new drugs, several strategies can be adopted, like database screening, either by *in silico* virtual screening or by *in vivo* functional assay on bacteria, combined with a classical structural drug design approach. Whatever the approach, it requires to use controls to estimate the benefit of the developed molecules compared to the ones previously developed and to current treatments. One of the admitted controls to evaluate the effect of a molecule as an efficient efflux pump inhibitor (EPI) is the use of the peptide inhibitor Phe-Arg-β-naphthylamide (PAβN). This molecule results from a screening for EPIs performed on *P. aeruginosa* in the presence of levofloxacin, with molecules issued from a Japanese pharmaceutical industry collection (Renau et al., 1999). PAβN has been further characterized for its ability to inhibit resistance of levofloxacin in different mutated strains of *P. aeruginosa* that overexpress or lack the MexAB-OprM, MexCD-OprJ or MexEF-OprN pumps, in order to precise its target (Lomovskaya et al., 2001). From MIC measurements of levofloxacin in presence of PAβN, and accumulation experiments performed with a fluorescent probe, Lomovskaya et al. (2001) proved PAβN was targeting the RND pumps of *P. aeruginosa* making it an interesting candidate as EPI. This interesting result was extended to other antibiotics from the fluoroquinolone family (Coban et al., 2004). However, despite these very interesting and positive results, PAβN has never been introduced in clinical practice as it was found to be toxic (Renau et al., 2003), even if it is difficult to find clear information on this point in the literature. Nevertheless, it is admitted to use it as a control for drug developments targeting RND efflux pumps (Yu et al., 2025; EUCAST, 2026), the literature being too extensive on its use to cite all of them.

In this work, for the future development of new EPIs, that requires the use of a validated reference, it was decided to establish the use limitations of PAβN for each envisaged experimental approach. -We determined PAβN concentrations’ limits when used as an adjuvant in combination with antibiotics on several *P. aeruginosa* strains, and its membranous envelope perturbation concentration limits. -We re-analyzed PAβN toxicity to obtain reference concentrations on the four different human cells used in our project. The values surprisingly revealed to be lower than expected, pushing us to further characterization. Consequently, the toxicity of β-naphthylamine was also investigated to assess whether the β-naphthylamide moiety could contribute to PAβN toxicity, under the hypothesis that PAβN may undergo physiological proteolysis. The use of this carcinogen molecule (Czubacka and Czerczak, 2020; Habil et al., 2022), was for setting the high toxicology limit for comparison of newly developed EPIs. -To complete the required information for a drug-design approach, and to understand how PAβN can block RND transporters at a molecular level, solving structures of RND transporters in complex with PAβN was necessary. One structure already exists in the PDB of PAβN solved with AcrB, the *Escherichia coli* homologue of MexB, at 3.6Å resolution (PDB code 1T9Y (Yu et al., 2005)). Nevertheless, this structure has been solved in the crystallographic space group H32 in which the trimer is formed by a crystallographic 3-fold axis, forcing the trimer to be symmetric. This configuration prevents the conformation changes required for the efflux of molecules, which could explain why the PAβN was present close to the entry of the efflux pathway instead of in the cavities, pushing us to determine new structures. Consequently, we solved several structures of MexB in complex with PAβN, by crystallography or by cryo-EM. These structures highlighted interesting information on the way PAβN interacts with this efflux pump transporter, suggesting the presence of different resting zones along the efflux pathway to take into account for the development of future EPIs by drug-design.

## 2 Methods

### 2.1 Minimum inhibitory concentration determination

Minimum inhibitory concentrations (MICs) were determined by broth microdilution in cation-adjusted Mueller–Hinton broth (CAMHB), following EUCAST recommendations with minor experimental adaptations. Wild-type PA14 *P. aeruginosa* (PA14-wt) (Liberati et al., 2006), PA14 Δ*mexAB* (PA14-ΔAB) (Tetard et al., 2019) and PA14 Δ*nalC* (PA14-AB+) (Tetard et al., 2022) strains were used for this study. Since the deletion of the expression regulator *nalC* results in MexAB-OprM overproduction, the PA14 Δ*nalC* strain refers to PA14-AB+ (Cao et al., 2004). Bacterial suspensions were prepared from overnight cultures, adjusted to 0.5 McFarland, and diluted in CAMHB to obtain an inoculum of approximately 5 × 10^5^ CFU/mL per well. Assays were performed in clear-bottom 96-well microplates (TPP 92697) in a final volume of 200 µL per well. Several antipseudomonal β-lactams that are widely used in a clinical context and are known to be good substrates of MexAB-OprM efflux pump (Masuda et al., 2000) were chosen as antibiotics, alone or in combination with PAβN. These include: aztreonam, a monobactam; ceftazidime, a cephalosporin; and ticarcillin, a carboxypenicillin. The concentrations of the antibiotics were prepared using the classical two-fold serial dilution method. PAβN was added at three final concentrations, 0, 50 or 100 μg/mL for comparison. These concentrations were selected as they fall within the range commonly used in efflux inhibition assays (EUCAST, 2026) and did not significantly affect bacterial growth under our experimental conditions. As a control, bacterial growth experiments were performed under the same conditions, but without antibiotics. MICs were estimated as the lowest antibiotic concentration showing no growth. In addition, PAβN was added at various concentrations ranging from 100 to 800 μg/mL to estimate its potential viability perturbation effect. The plates were incubated at 37 °C for 20 h without shaking. After this time, bacterial growth was measured at OD600 using a Thermo Scientific Multiskan FC microplate reader.

### 2.2 Toxicity evaluation of PAβN and β-naphthylamine on different cell lines

#### 2.2.1 General conditions

##### 2.2.1.1 Cell lines and culture conditions

Human bronchial epithelial cells (BEAS-2B), human alveolar epithelial carcinoma cells (A549), human embryonic kidney cells (HEK293), and human hepatocellular carcinoma cells (HepG2) were used to evaluate the cytotoxic effects of PAβN and β-naphthylamine. These cell lines were selected to represent different human tissues potentially exposed to xenobiotics, including pulmonary, renal, and hepatic models, as well as both non-tumoral and tumoral cellular phenotypes.

BEAS-2B, A549 and HEK293 cells were cultured in Dulbecco’s Modified Eagle Medium (DMEM), whereas HepG2 cells were maintained in Minimum Essential Medium (MEM). Culture media were supplemented with 10% fetal bovine serum (FBS), 1% L-glutamine, and 0.5% penicillin–streptomycin. Cells were maintained at 37 °C in a humidified incubator containing 5% CO_2_.

Cells were detached using trypsin–EDTA solution after overnight adherence and seeded into 96-well microplates at densities of 5 × 10^4^ cells/mL for BEAS-2B and A549 cells, 1.2 × 10^5^ cells/mL for HEK293 cells, and 2 × 10^5^ cells/mL for HepG2 cells.

##### 2.2.1.2 Conditions of cells exposure to PAβN or β-naphthylamine

A stock solution of PAβN (Phe-Arg β-naphthylamide dihydrochloride, purity ≥98%, Sigma-Aldrich, Saint Louis, MO, United States) was prepared in dimethyl sulfoxide (DMSO) at 100 mg/mL and subsequently diluted in culture medium supplemented with 2.5% fetal bovine serum. A stock solution of β-naphthylamine (purity ≥95%, Supelco, Sigma-Aldrich, Saint Louis, MO, United States) was prepared at 100 mg/mL under the same conditions. Reduced serum conditions were used to minimize serum-related interference with compound bioavailability during exposure experiments. Cells were exposed to increasing concentrations of PAβN ranging from 50 to 750 μg/mL (approximately 96 μM to 1.44 mM), while HepG2 cells were additionally exposed to 1,000 μg/mL PAβN (approximately 1.93 mM) or β-naphthylamine ranging from 0.1 to 1,000 μg/mL (approximately 0.7 µM–7 mM). Concentration ranges were selected to characterize concentration-dependent cellular responses and investigate potential mechanisms of toxicity rather than to reproduce physiological exposure conditions. The highest concentrations tested were constrained by the aqueous solubility of PAβN under cell culture conditions. Indeed, at higher concentrations, PAβN progressively formed particulate material after dilution into the cell culture medium, limiting the use of higher concentrations under our experimental conditions. In all experiments, the final concentration of DMSO did not exceed 1% (v/v). Cells were exposed for 24 h prior to LDH cytotoxicity, cell viability, and caspase activity analyses. Intracellular ROS production, mitochondrial membrane potential, and ATP levels were additionally assessed at different time points following exposure in order to characterize early cellular responses and mitochondrial dysfunction induced by the two compounds, PAβN or β-naphthylamine.

##### 2.2.1.3 Statistical analysis

All experiments were independently performed at least three times. Data are presented as mean ± standard deviation (SD). Statistical analyses were performed using GraphPad Prism software (version 8.0.1, GraphPad Software, San Diego, CA, United States). Data distribution normality was assessed prior to statistical analysis using the Shapiro–Wilk test. For normally distributed data, statistical comparisons were performed using parametric tests, including one-way analysis of variance (ANOVA) followed by Dunnett’s multiple comparison test. For non-normally distributed data, non-parametric analyses were performed using the Kruskal–Wallis test followed by Dunn’s multiple comparison test, as appropriate. Comparisons between two independent groups (1 h vs. 24 h at the same concentration) were performed using an unpaired Student’s t-test. A p-value <0.05 was considered statistically significant. Statistical significance was defined as follows: *p < 0.05, **p < 0.01, ***p < 0.001, and ****p < 0.0001.

#### 2.2.2 Toxicity evaluation

##### 2.2.2.1 Cytotoxicity assessment by LDH

Cell membrane integrity was evaluated using the CyQUANT™ LDH Cytotoxicity Assay kit (Thermo Fisher Scientific, Waltham, MA, United States), according to the manufacturer’s instructions. Following PAβN or β-naphthylamine exposure, culture supernatants were collected and incubated with the reaction mixture provided in the kit. Absorbance was measured using a Spark® multimode microplate reader (Tecan, Männedorf, Switzerland) at 490 nm with background correction at 680 nm. Cytotoxicity was expressed relative to untreated control cells.

##### 2.2.2.2 Cell viability assay (alamar blue)

Cell viability was assessed using the Alamar Blue assay, which evaluates cellular metabolic activity through the reduction of resazurin to the fluorescent compound resorufin by metabolically active cells. Following PAβN or β-naphthylamine exposure, cells were incubated for 6 h at 37 °C with Alamar Blue reagent prepared from a 0.1 mg/mL stock solution and diluted 1:11 in culture medium supplemented with 2.5% fetal bovine serum. Fluorescence was measured using a Spark® multimode microplate reader (Tecan, Männedorf, Switzerland) with excitation and emission wavelengths of 535 nm and 600 nm, respectively. Cell viability was expressed relative to untreated control cells.

##### 2.2.2.3 Cell counting and membrane integrity analysis

Cell number was determined following 24 h PAβN exposure. Cells were detached using trypsin-EDTA and manually counted using a Thoma hemocytometer.

Cell membrane permeability was additionally evaluated using propidium iodide (PI) staining (Invitrogen, Thermo Fisher Scientific, Waltham, MA, United States). Following PAβN exposure, cells were incubated with PI and fluorescence was measured using a Spark® multimode microplate reader (Tecan, Männedorf, Switzerland) with excitation and emission wavelengths of 535 nm and 620 nm, respectively. PI uptake was used as an indicator of membrane damage and loss of cell viability.

##### 2.2.2.4 Intracellular ROS measurement (H2DCF-DA)

Intracellular reactive oxygen species (ROS) production was assessed using the H2DCF-DA fluorescent probe (Invitrogen, Thermo Fisher Scientific, Waltham, MA, United States). Following intracellular deacetylation by cellular esterases and oxidation by reactive oxygen species, the probe generates the fluorescent compound dichlorofluorescein (DCF). Cells were exposed to PAβN for 1, 3, 6 and 24 h or to β-naphthylamine for 1h and 24h prior to ROS measurement. Cells were then incubated with H2DCF-DA for 20 min at 37 °C in the dark. Fluorescence was measured using a Spark® multimode microplate reader (Tecan, Männedorf, Switzerland) with excitation and emission wavelengths of 485 nm and 535 nm, respectively. ROS production was expressed relative to untreated control cells.

##### 2.2.2.5 Mitochondrial membrane potential analysis (JC-1)

Mitochondrial membrane potential (ΔΨm) was evaluated using the JC-1 fluorescent probe (Invitrogen, Thermo Fisher Scientific, Waltham, MA, United States). In healthy mitochondria with high membrane potential, JC-1 forms red fluorescent aggregates, whereas under mitochondrial depolarization conditions, the probe remains in its green fluorescent monomeric form. Following PAβN or β-naphthylamine exposure, cells were incubated with JC-1 probe for 15 min at 37 °C in the dark. Fluorescence was measured using a Spark® multimode microplate reader (Tecan, Männedorf, Switzerland) for both green and red emissions. Mitochondrial membrane potential alterations were expressed as the ratio of red to green fluorescence intensity. Changes in ΔΨm were assessed at 1 and 24 h following PAβN or β-naphthylamine exposure.

##### 2.2.2.6 Intracellular ATP quantification

Intracellular ATP levels were quantified using the CellTiter-Glo® 2.0 Cell Viability Assay kit (Promega, Madison, WI, United States), according to the manufacturer’s instructions. This luminescence-based assay measures cellular ATP content as an indicator of metabolically active cells. Cells were exposed to PAβN or β-naphthylamine for 1 h and 24 h prior to ATP quantification. An equal volume of CellTiter-Glo® reagent was added to each well and luminescence was measured using a Spark® multimode microplate reader (Tecan, Männedorf, Switzerland). ATP levels were expressed relative to untreated control cells.

##### 2.2.2.7 Caspase activity assays

Caspase-3/7, caspase-8, and caspase-9 activities were evaluated using Caspase-Glo® assay kits (Promega, Madison, WI, United States), according to the manufacturer’s instructions. These luminescent assays measure the activation of executioner caspases (caspase-3/7), extrinsic apoptotic pathway-associated caspase-8, and mitochondria-associated intrinsic apoptotic pathway caspase-9. Cells were exposed to PAβN or β-naphthylamine for 24 h prior to caspase activity analysis. Following reagent addition, plates were incubated for 1 h at room temperature and luminescence was measured using a Spark® multimode microplate reader (Tecan, Männedorf, Switzerland). Caspase activities were expressed relative to untreated control cells.

### 2.3 Purification of MexB

For the structural biology applications, the mutated form of MexB (MexB-D407N) that abrogates the proton transport, later called MexB, was used instead of the wild-type as the purification yield is higher for this construct. The *P. aeruginosa* MexB multidrug transporter was cloned into the pET41a expression vector in frame with a 8×His tag at the C-terminus. The plasmid was transformed into *E. coli* C43 (DE3)*ΔacrAB* cells, which harbor a deletion of the chromosomal *acrAB* genes (personal gift from MK. Pos (Goethe-University Frankfurt, Germany)). Cells were grown in 6 L of a Terrific Broth medium supplemented with 50 μg/mL kanamycin at 37 °C. When the OD_600_ reached 0.6, the expression of MexB was induced with 1 mM isopropyl-β-D-thiogalactopyranoside. Cells were then harvested after overnight induction at 18 °C at 10,000 g for 15 min at 4 °C. The collected bacterial cells were resuspended in lysis buffer (20 mM Bis-Tris [pH 7.4], 200 mM sodium chloride and a tablet of SigmaFAST™ Protease Inhibitor Cocktail) and disrupted with a Cell Disruptor. The membrane fraction was collected after an ultracentrifugation at 100,000 × g for 1h at 4 °C, then solubilized in 1.5% (wt/vol) n-dodecyl-β-D-maltoside (DDM). Insoluble material was removed by ultracentrifugation at 145,000 × g for 1 h, and the supernatant was loaded on a Ni-NTA (Sigma) affinity column with a buffer containing (20 mM Bis-Tris pH 7.4, 200 mM sodium chloride, 10% glycerol, 10 mM imidazole pH 8 and 0.05% DDM) and then eluted with the same buffer containing 300 mM imidazole pH 8. The purified protein was concentrated with 100 kDa Amicon and injected in a Superose 6 column (Cytiva) equilibrated with 20 mM HEPES pH 7.5, 50 mM sodium chloride, and 0.03% DDM. The purified protein was aliquoted and kept at −80 °C until use.

### 2.4 Resolution of the structure of MexB in complex with PAβN by crystallography

#### 2.4.1 Crystallization of MexB and of the MexB–PAβN complex

Prior to crystallization, 1 mg/mL of purified MexB were submitted to overnight limited proteolysis with bovine trypsin, at protease to MexB ratio of 1:1,000 (w/w), in buffer 20 mM HEPES pH 7.5, 50 mM NaCl, 0.03% DDM (w/v). Proteolysis was quenched by the addition of 1 mM TLCK (tosyl-L-lysine chloromethyl ketone). MexB was then purified by gel filtration with Superose 6 column (Cytiva) and concentrated up to 10 mg/mL for crystallogenesis. Crystals of MexB were obtained using the sitting-drop vapor diffusion method at 20 °C by mixing equal volumes of purified protein and reservoir solution. Crystallization conditions consisted of 18%–20% PEG 400 (v/v) and 100 mM sodium acetate at pH 4.5. Crystals appeared after 2 days and reached maximal dimensions of 50–100 μm within 1 week. To obtain the structure of MexB with PAβN, MexB crystals were soaked in reservoir solution supplemented with the ligand PAβN at 1–2 mM for 10 min at 20 °C. Prior to data collection, crystals were cryoprotected with 25% PEG 400 and flash-cooled in liquid nitrogen.

#### 2.4.2 X-ray data collection and structure determination

Diffraction data were collected at the X-ray beamline ID30A3 of the European Synchrotron Radiation Facility (ESRF, Grenoble) at a wavelength of 0.9677 Å and a temperature of 100 K. Data were processed and scaled using XDS (Kabsch, 2010) and AIMLESS (Agirre et al., 2023). The structure was solved by molecular replacement using PHASER (McCoy et al., 2007) with the previously reported MexB structure (PDB ID: 2V50) as the search model. Iterative cycles of model building and refinement were carried out using COOT (Emsley and Cowtan, 2004) and PHENIX (Adams et al., 2010), respectively. Structural validation was performed using MolProbity. Data collection and refinement statistics are summarized in Supplementary Table S1.

#### 2.4.3 Structural analysis

Structural figures and interaction analyses were generated using PyMOL (DeLano, 2002). Protein–ligand interactions were analyzed by measuring hydrogen bonds, hydrophobic contacts, and aromatic stacking interactions within a cutoff distance of 4.0 Å.

### 2.5 Resolution of the structure of MexB in complex with PAβN by cryo-electron microscopy

#### 2.5.1 Preparation of the MexB/PAβN complex in nanodiscs

To stabilize the protein in a more natural lipid environment, MexB was reconstituted in nanodisc, as previously described (Wehbi et al., 2025) with minor modifications. MexB was mixed with POPC lipids and with the membrane scaffold protein (MSP) 1E3D1 (MSP1E3D1). The mixture of 17.3 µM MexB, 34.6 µM MSP1E3D1, 1,222 µM POPC corresponding to a ratio (1:2:70) was performed on ice for 15 min. A solution of pre-washed Bio-beads (Bio-Rad) was added in an amount equivalent to 50 times the total mass of DDM, in order to remove the detergent. The resultant mixture was incubated overnight at 4 °C. The Bio-beads were removed by a centrifugation at 14,000 g for 5 min at 4 °C. The protein-nanodisc solution was further purified using a Superose 6 column (Cytiva) equilibrated with 10 mM Tris-HCl pH 7.4 and 100 mM NaCl. Fractions corresponding to the size of the complex MexB/nanodisc were collected for cryo-EM sample preparation. Purified fractions containing MexB reconstituted in nanodiscs were incubated overnight at 4 °C with PAβN at an optimized final concentration of 30 µM. Prior to cryo-EM grid preparation, the sample was diluted in the MexB nanodisc buffer to a final MexB concentration of 400 μg/mL (≈1.2 μM). The PAβN concentration of 30 µM was first chosen to be above the low-micromolar affinity range suggested by our preliminary binding experiments (data not shown), and consistent with the reported affinity of PAβN for the homologous RND transporter AcrB (Kd = 15.72 ± 3.0 µM) (Mowla et al., 2018). During grid optimization, PAβN concentrations above 30 µM led to nanodisc aggregation on the grids, as visualized by negative staining electronic microscopy, and poor sample distribution across the vitrified area, preventing reliable data acquisition.

#### 2.5.2 Preparation of the grids, data collection and processing

Cryo-EM grid preparation was performed on a Quantifoil UltrAuFoil R1.2/1.3 300-mesh gold grid. After glow discharge for 30 s at 15 mA, 4 µL of the nanodisc-reconstituted MexB sample incubated with PAβN was applied to the grids and vitrified using a Leica EM GP2 plunge freezer at 8 °C and 90% humidity, with a blotting time of 1.5 s. The grids were first screened on a Glacios microscope (Curie platform CurieCoreTech, Paris) and subsequently selected for high-resolution data collection. High resolution Cryo-EM data were collected at the POLARIS cryo-EM facility at SOLEIL on a Thermo Fisher Scientific Titan Krios G4 microscope operated at 300 kV and equipped with a Falcon 4i direct electron detector and a Selectris X energy filter. A total of 10,483 movies were recorded. Cryo-EM data processing was performed in cryoSPARC v4.7.1 (Punjani et al., 2017). Movies were motion-corrected and dose-weighted using Patch Motion Correction, followed by CTF estimation with Patch CTF Estimation. An initial set of approximately 1.5 million particles was generated using the Blob Picker. Extracted particles were subjected to five rounds of 2D classification. Classes displaying well-defined MexB particles were retained, yielding to 886,949 particles, which were then used to generate three *ab initio* reconstructions. The best-resolved volume was refined by homogeneous refinement followed by non-uniform refinement. CTF refinement and a final round of non-uniform refinement yielded a reconstruction at an overall resolution of 2.31 Å, estimated using the gold-standard FSC = 0.143 criterion. The final map was reconstructed from 592,436 particles. (Scheres and Chen, 2012).

#### 2.5.3 Structure resolution

An in-house MexB atomic model derived from a previously refined unpublished structure was used as the initial model. The model was rigid-body fitted into the cryo-EM density using UCSF Chimera (Pettersen et al., 2004) and manually adjusted in Coot (Emsley et al., 2010). PAβN was modeled into the density assigned to the ligand, with restraints generated using eLBOW in Phenix (Moriarty et al., 2009). The structure was refined by real-space refinement in Phenix, followed by iterative manual corrections in Coot. The final model was inspected and validated using Phenix and Coot.

## 3 Results

### 3.1 Effect of PAβN on the growth and cellular envelop integrity of *Pseudomonas aeruginosa* in the presence or not of β-lactam antibiotics

The ability of PAβN to potentiate the activity of three β-lactams (aztreonam, ceftazidime, and ticarcillin) against PA14-wt, PA14-ΔAB and PA14-AB + strains was tested (Table 1). In the absence of PAβN, PA14-AB + displayed higher MICs compared to the wild-type strain for the three tested β-lactams, consistent with the increased expression of the MexAB-OprM efflux pump. Conversely, PA14-ΔAB showed lower MICs, in agreement with the loss of the constitutive efflux pump MexAB-OprM. Addition of PAβN reduced the MIC values in PA14-wt and PA14-AB+, with the strongest effects observed at 100 μg/mL (192 µM). In PA14-AB+, PAβN reduced aztreonam and ceftazidime MICs by 4-fold, whereas the MIC for ticarcillin was decreased by 8-fold. In contrast, no reduction of the MIC was observed in PA14-ΔAB, supporting a MexAB-OprM-associated effect of PAβN under these conditions.

**TABLE 1:** MICs of several !-lactam antibiotics with or without PA!N for the Pseudomonas aeruginosa PA14 strain and its derivatives. MIC values are expressed in μg/mL. Added PA!N concentrations are indicated. PA14-“AB and PA14-AB + correspond, respectively, to a mexAB-deleted mutant of PA14 and a MexABOprM overproducer (due to a mutation in nalC) of PA14. Fold changes are indicated in respect to the PA14-wt without PA!N value indicated in bold.

​	​	MICs of the three tested antibiotics in the presence of increasing amount of PAbN (μg/mL) (fold change compared to PA14-wt)		
Antibiotic	Strain	+ PAβN 0	+ PAβN 50 μg/mL	+ PAβN 100 μg/mL
Aztreonam	PA14-wt	**4**	2 (/2)	1 (/4)
	PA14-ΔAB	1 (/4)	1 (/4)	1 (/4)
	PA14-AB+	16 (x4)	8 (x2)	4 (=)
Ceftazidime	PA14-wt	**2**	1 (/2)	1 (/2)
	PA14-ΔAB	0.5 (/4)	0.5 (/4)	0.5 (/4)
	PA14-AB+	8 (x4)	4 (x2)	2 (=)
Ticarcilline	PA14-wt	**32**	16 (/2)	4 (/8)
	PA14-ΔAB	8 (/4)	8 (/4)	8 (/4)
	PA14-AB+	64 (x2)	16 (/2)	8 (/4)

In the conditions used for the MICs determination, the controls performed with PAβN alone showed no significant growth perturbation at 100 μg/mL, whereas a growth perturbation was observed at 200 μg/mL, and a marked growth inhibition between 400 and 800 μg/mL. Together with the absence of MIC reduction, whatever the antibiotic, in the PA14-ΔAB strain, these controls indicate that the observed effect of antibiotic potentiation by PAβN when used up to 100 μg/mL, is mainly associated with MexAB-OprM activity rather than a nonspecific antibacterial effect of PAβN.

### 3.2 Toxicity evaluation of PAβN and β-naphthylamine on different cell lines

The effects of PAβN and β-naphthylamine exposure were investigated in a panel of human cell lines, including cancer-derived (A549, HepG2), and immortalized non-tumor cell lines (BEAS-2B, HEK293), to evaluate cytotoxic and mechanistic responses across different cellular contexts.

#### 3.2.1 Cell viability and cytotoxicity assessment

The effects of PAβN and β-naphthylamine on cell metabolic activity were assessed using the Alamar Blue assay after 24 h of exposure (Figure 1). PAβN had limited effects on metabolic activity, with a decrease in Alamar Blue reduction observed only at the highest concentrations tested in BEAS-2B and HEK293 cells. In contrast, β-naphthylamine produced a more pronounced reduction in Alamar Blue reduction, although the magnitude of the response varied between cell lines, with the strongest effects observed in BEAS-2B and HEK293 cells (Figures 1D–F). Indeed, at 500 μg/mL (3,492.6 µM), cell viability decreased to 23% in HEK293 cells and 34% in BEAS-2B cells, while A549 and HepG2 cells retained 55% and 72% viability, respectively, at the same concentration.

**FIGURE 1:**
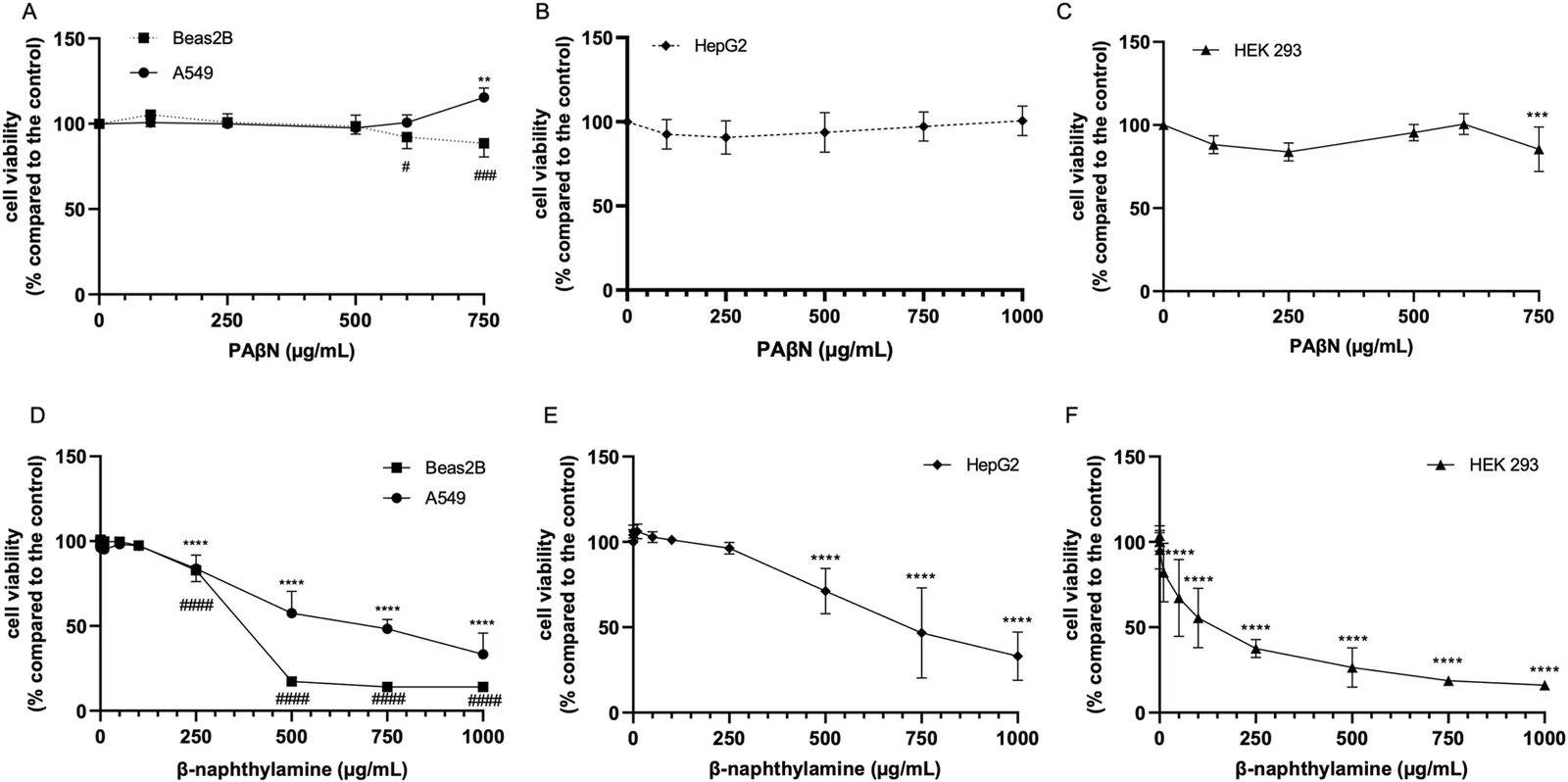
Effect of PAβN and β-naphthylamine on cell viability in human cell lines. **(A–C)** PAβN and **(D–F)** β-naphthylamine. Cells were exposed to PAβN or β-naphthylamine at the indicated concentrations for 24 h, with each concentration tested in a separate experimental condition. Correspondence between mass and molar concentrations is provided in Supplementary Table S2. Cell viability was assessed using the resazurin (Alamar Blue) assay. Data are presented as mean ± SD from three independent experiments (n = 3). Statistical analyses were performed using one-way ANOVA followed by Dunnett’s multiple comparisons test, with each concentration compared with the corresponding untreated control (0 μg/mL). Symbols indicate cell lines: A549 (●), BEAS-2B (■), HepG2 (◆), and HEK293 (▲). Statistical significance is indicated by *, **, ***, and **** for p < 0.05, p < 0.01, p < 0.001, and p < 0.0001, respectively; #, ##, ###, and #### indicate the same significance levels for the second cell line shown on the same graph.

To further assess cytotoxicity, lactate dehydrogenase (LDH) release was measured in cell culture supernatants after 24 h of treatment (Figure 2). LDH release reflects loss of plasma membrane integrity and cellular damage. PAβN induced a modest increase in LDH release across all cell lines, reaching approximately 1.5–2-fold at the highest concentrations tested (750–1,000 μg/mL; 1,443.8–1925.0 µM). β-naphthylamine caused a marked, dose-dependent increase in LDH release. The strongest effects were observed in HepG2 and HEK293 cells, with approximately 3–4-fold increases at 1,000 μg/mL (6,985.1 µM). A549 and BEAS-2B cells showed more moderate increases, ranging from approximately 2–3-fold under the same conditions.

**FIGURE 2:**
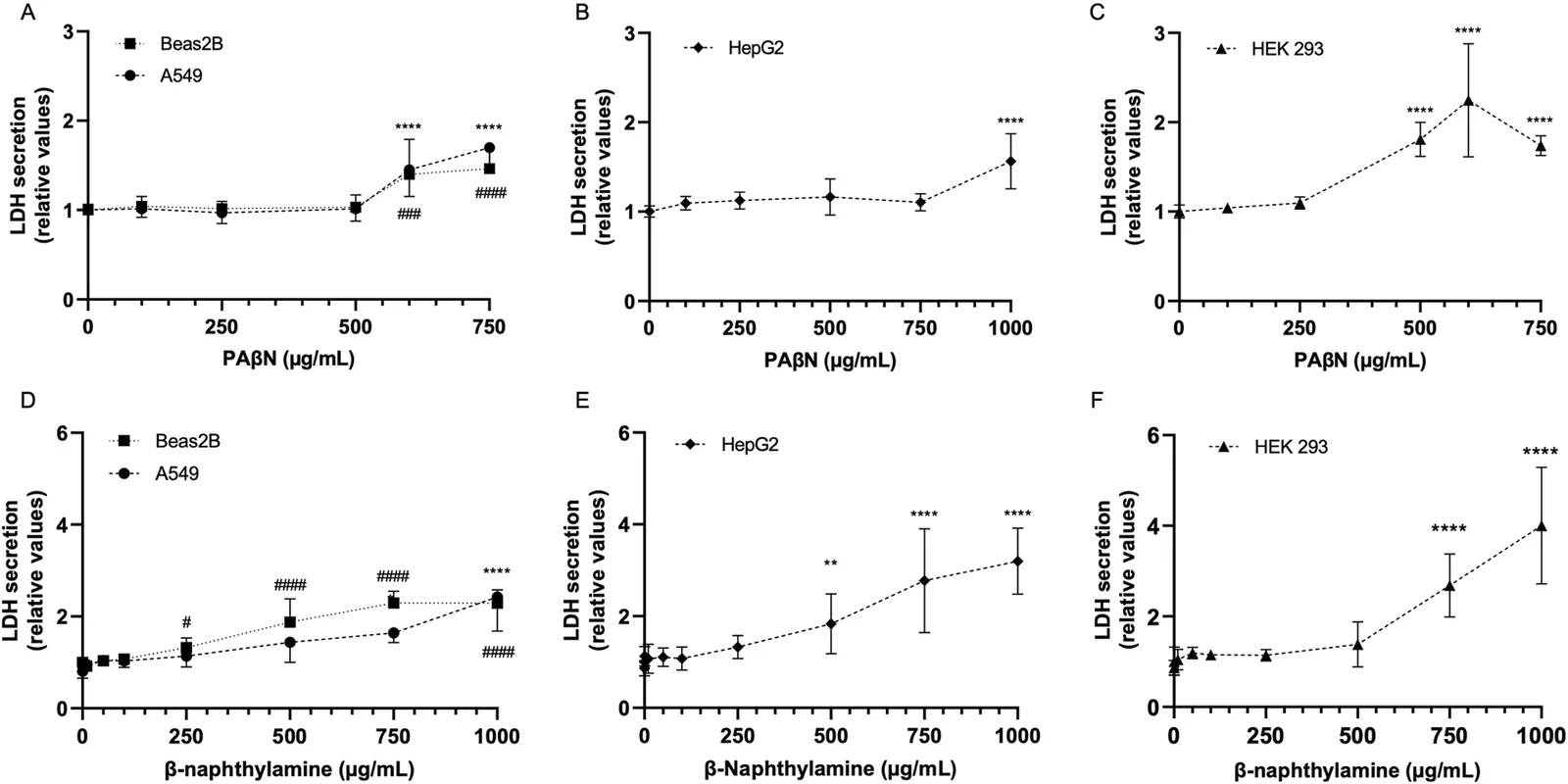
PAβN and β-naphthylamine induce concentration-dependent cytotoxicity in human cell lines. **(A–C)** PAβN and **(D–F)** β-naphthylamine. Cells were exposed to PAβN or β-naphthylamine at the indicated concentrations for 24 h, with each concentration tested in a separate experimental condition. Correspondence between mass and molar concentrations is provided in Supplementary Table S2. Cytotoxicity was assessed using the LDH assay. Data are presented as mean ± SD from three independent experiments (n = 5). Statistical analyses were performed using one-way ANOVA followed by Dunnett’s multiple comparisons test, with each concentration compared with the corresponding untreated control (0 μg/mL). Symbols indicate cell lines: A549 (●), BEAS-2B (■), HepG2 (◆), and HEK293 (▲). Statistical significance is indicated by *, **, ***, and **** for p < 0.05, p < 0.01, p < 0.001, and p < 0.0001, respectively; #, ##, ###, and #### indicate the same significance levels for the second cell line shown on the same graph.

To further characterize the cellular effects of PAβN, cell number and plasma membrane integrity were assessed using complementary approaches (Supplementary Figure S1). Cell number decreased at concentrations ≥500 μg/mL (962.5 µM) in BEAS-2B and A549 cells and at concentrations ≥250 μg/mL (481.3 µM) in HEK293 cells, whereas no clear decrease was observed in HepG2 cells. In parallel, PI uptake showed only a limited increase at high PAβN concentrations in BEAS-2B and A549 cells, whereas a more pronounced increase was observed in HEK293 cells.

Overall, PAβN produced relatively limited effects on metabolic activity and plasma membrane integrity, with responses varying between cell lines. In contrast, β-naphthylamine caused a more pronounced reduction in metabolic activity, particularly in BEAS-2B and HEK293 cells, indicating distinct cellular response profiles between the two compounds.

#### 3.2.2 PAβN and β-naphthylamine differentially affect oxidative stress and mitochondrial function

##### 3.2.2.1 PAβN and β-naphthylamine induce distinct ROS responses

To investigate whether oxidative stress contributes to the differential cellular responses induced by PAβN and β-naphthylamine, intracellular ROS production was measured after increasing time exposures (Figure 3). PAβN induced only limited ROS production during the early exposure periods (1–6 h) in A549, BEAS-2B, and HEK293 cells, with significant increases observed only at the highest tested concentration (Figure 3; Supplementary Figure S2). In contrast, a marked concentration-dependent increase in ROS production was observed after 24 h of exposure in these 3 cell lines. HepG2 cells appeared more sensitive to PAβN-induced oxidative stress, exhibiting significantly elevated ROS levels from 100 μg/mL (192.5 µM) at all exposure times tested.

**FIGURE 3:**
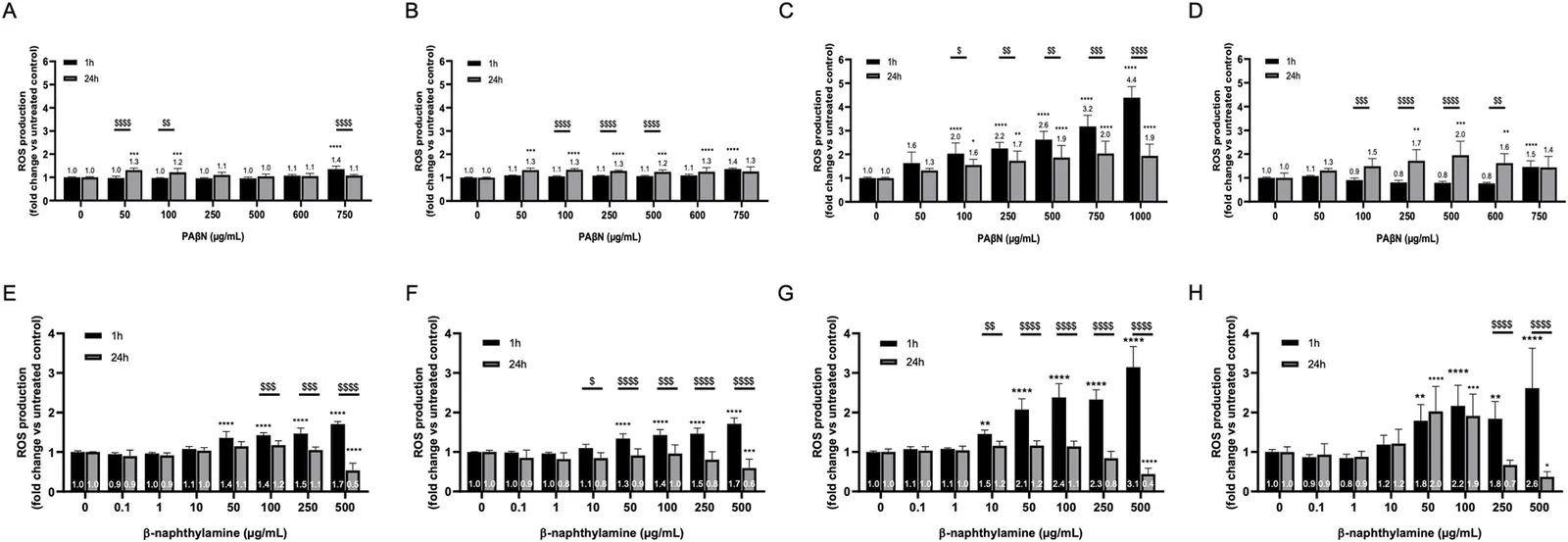
Time-dependent ROS production induced by PAβN and β-naphthylamine. Intracellular ROS levels were measured in BEAS-2B **(A,E)**, A549 **(B,F)**, HepG2 **(C,G)** and HEK293 **(D,H)** cells using the H_2_DCFH-DA assay. **(A–D)** Cells were exposed to PAβN, **(E–H)** Cells were exposed to β-naphthylamine for 1 h and 24 h. Data are expressed as fold change relative to untreated control cells and presented as mean ± SD (n = 3). Statistical significance: * compared with the corresponding untreated control (0 μg/mL) (*p < 0.05, **p < 0.01, ***p < 0.001, ****p < 0.0001); § compared with the corresponding 1 h treatment at the same concentration (§p < 0.05, §§p < 0.01, §§§p < 0.001).

In contrast, β-naphthylamine triggered a rapid and pronounced increase in ROS production after 1 h of exposure in all cell lines (Figure 3). However, ROS levels were significantly reduced at the highest concentrations after 24 h. This decrease coincides with the marked loss of metabolic activity and the increased cytotoxicity observed in the Alamar Blue and LDH assays, respectively (Figures 1, 2), suggesting that the reduction in ROS signal may reflect the extensive loss of viable cells at these concentrations.

##### 3.2.2.2 PAβN and β-naphthylamine differentially alter mitochondrial membrane potential

To investigate whether mitochondrial dysfunction contributes to the cellular effects of PAβN and β-naphthylamine, mitochondrial membrane potential (Δψm) was assessed using the JC-1 assay (Figure 4).

**FIGURE 4:**
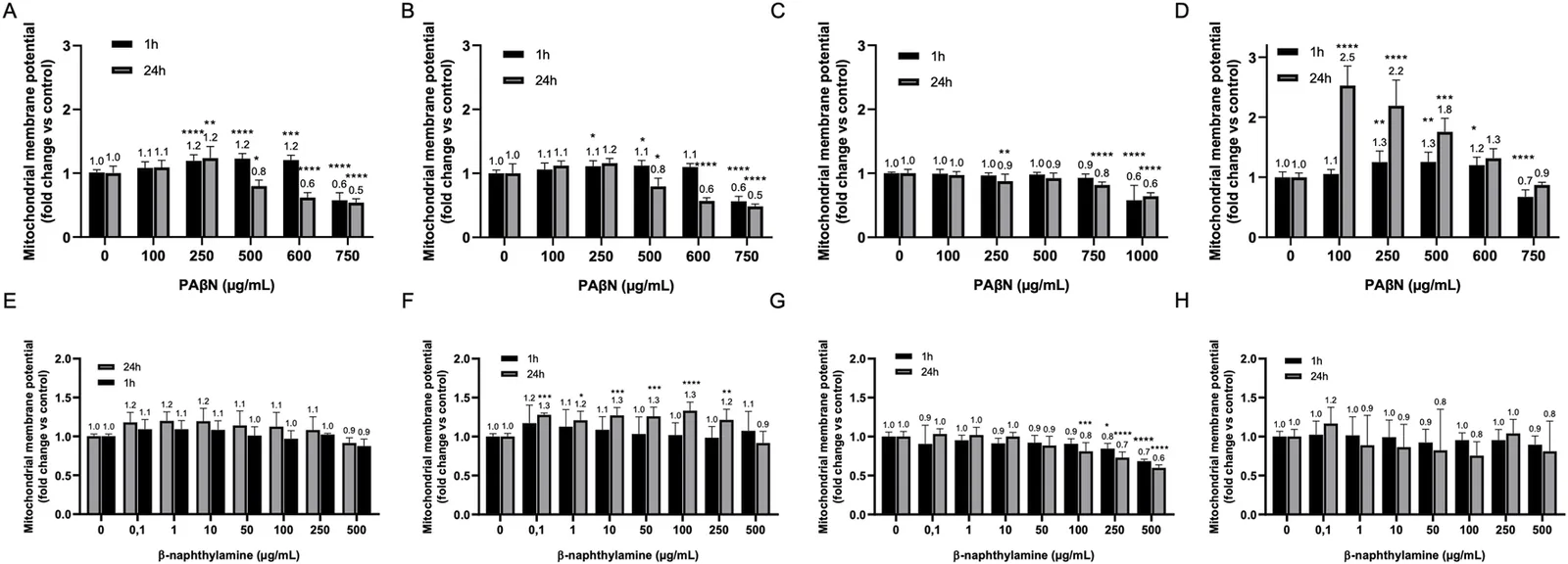
Biphasic and cell line-specific effects of PAβN and β-naphthylamine on mitochondrial membrane potential (Δψm). Mitochondrial membrane potential (Δψm) was assessed using the JC-1 assay in BEAS-2B **(A,E)**, A549 **(B,F)**, HepG2 **(C,G)**, and HEK293 **(D,H)** cells following 1 h and 24 h exposure to **(A–D)** PAβN (0–750 μg/mL; approximately 0–1.44 mM) or **(E–H)** β-naphthylamine (0–500 μg/mL; approximately 0–3.49 mM). Data are expressed as the JC-1 red/green fluorescence ratio and presented as fold change relative to untreated control cells (mean ± SD, n = 3). An increase in the red/green fluorescence ratio indicates mitochondrial hyperpolarization, whereas a decrease indicates mitochondrial depolarization. Statistical significance was determined relative to the corresponding untreated control (*p < 0.05, **p < 0.01, ***p < 0.001, ****p < 0.0001).

PAβN induced a biphasic Δψm response in BEAS-2B, A549, and HEK293 cells (Figure 4). At low to intermediate concentrations (100–250 μg/mL; 192.5–481.3 µM), PAβN significantly increased Δψm after both 1 h and 24 h of exposure. In contrast, higher concentrations (500–750 μg/mL; 962.5–1,443.8 µM) induced a significant decrease in Δψm, indicative of mitochondrial depolarization and dysfunction. Notably, HEK293 cells displayed a marked and sustained increase in Δψm at 24 h despite elevated ROS production. This pattern may reflect an adaptive mitochondrial response to PAβN-induced oxidative stress. In HepG2 cells, PAβN induced mitochondrial depolarization only at high concentrations (750–1,000 μg/mL; 1,443.8–1925.0 µM), without a preceding hyperpolarization phase. This response was associated with the strong ROS production observed in this cell line.

β-naphthylamine also altered mitochondrial membrane potential in a cell type-dependent manner. In A549 cells, β-naphthylamine induced a significant mitochondrial hyperpolarization at 10–250 μg/mL (69.8–1746.0 µM) after 24 h of exposure, followed by significant depolarization at 500 μg/mL (3,492.6 µM). In HepG2 cells, β-naphthylamine induced mitochondrial depolarization at concentrations ranging from 100 to 500 μg/mL (698.4–3,492.6 µM) after 24 h. In contrast, no significant changes in Δψm were observed in BEAS-2B or HEK293 cells following β-naphthylamine treatment (Figure 4).

##### 3.2.2.3 Effects of PAβN and β-naphthylamine on cellular ATP levels

To further investigate the impact of PAβN and β-naphthylamine on cellular bioenergetics, intracellular ATP levels were measured after 1 h and 24 h of exposure (Figure 5).

**FIGURE 5:**
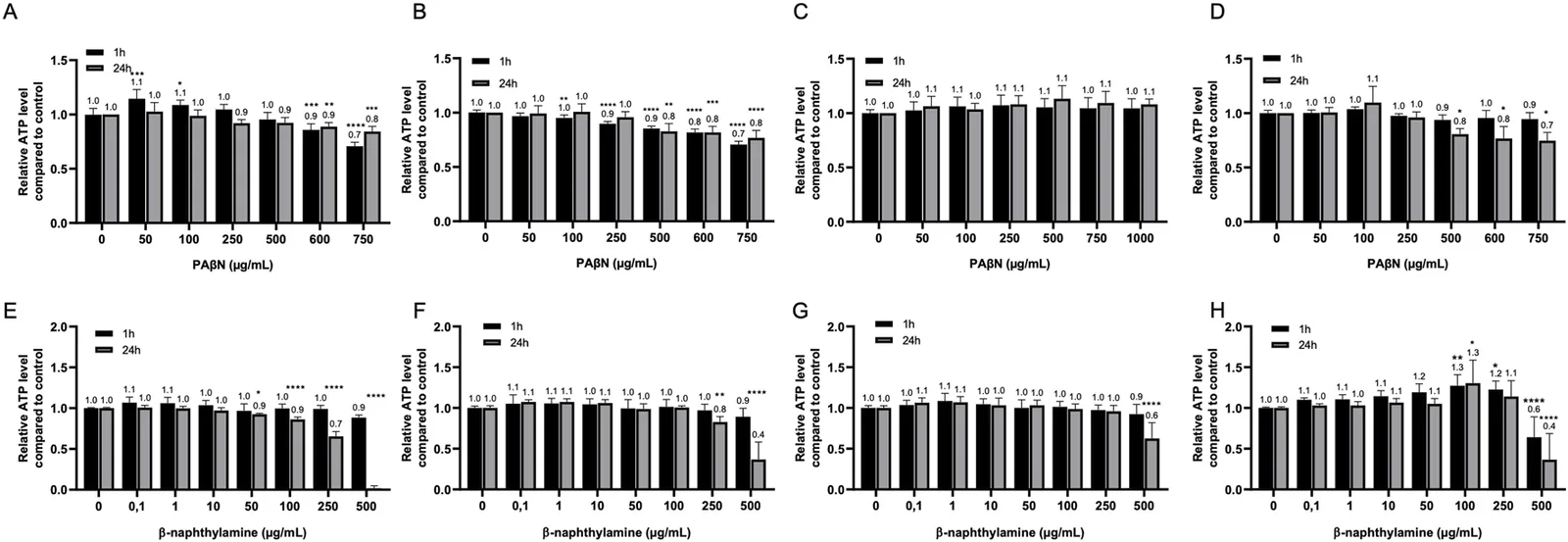
Differential effects of PAβN and β-naphthylamine on intracellular ATP levels in human cell lines. Intracellular ATP levels were measured in BEAS-2B **(A,E)**, A549 **(B,F)**, HepG2 **(C,G)** and HEK293 **(D,H)** cells following 1 h and 24 h exposure to **(A–D)** PAβN (0–1,000 μg/mL; approximately 0–1.9 mM) or **(E–H)** β-naphthylamine (0–500 μg/mL; approximately 0–3.5 mM). Data are expressed as fold change relative to untreated control cells and presented as mean ± SD (n = 3). Statistical significance was determined relative to the corresponding untreated control (*p < 0.05, **p < 0.01, ***p < 0.001, ****p < 0.0001).

PAβN induced a significant decrease in ATP levels after 1 h of exposure at the highest tested concentrations in A549 and BEAS-2B cells. In the two other cell lines, no significant changes were observed after 1 h exposure. However, after 24 h, a significant decrease in ATP levels was observed at the highest concentrations, consistent with mitochondrial membrane depolarization detected by the JC-1 assay in HEK293, A549, and BEAS-2B cells. No significant changes in ATP levels were observed in HepG2 cells.

β-naphthylamine induced dose- and cell line-dependent alterations in ATP levels. In A549 and BEAS-2B cells, β-naphthylamine caused a marked depletion of ATP after 24 h of exposure from 50 μg/mL (349.2 µM). In HEK293 cells, β-naphthylamine induced a biphasic response characterized by an increase in ATP levels at 100 μg/mL (698.4 µM), followed by a significant decrease at 500 μg/mL (3,492.6 µM). This pattern is consistent with an early adaptive response followed by mitochondrial dysfunction at higher concentrations. In HepG2 cells, ATP depletion was observed only at the highest concentration tested (500 μg/mL) after 24 h of exposure.

Together, ROS production, Δψm alterations, and ATP depletion indicate that both compounds disrupt mitochondrial homeostasis, although with distinct temporal and cell line-specific profiles.

#### 3.2.3 PAβN and β-naphthylamine differentially engage intrinsic and extrinsic apoptotic pathways

To investigate whether apoptosis contributes to the differential cytotoxic effects of PAβN and β-naphthylamine, the activities of caspase-3/7 (executioner caspase), caspase-8 (extrinsic initiator), and caspase-9 (intrinsic initiator) were measured in all cell lines after 24 h of exposure (Figure 6).

**FIGURE 6:**
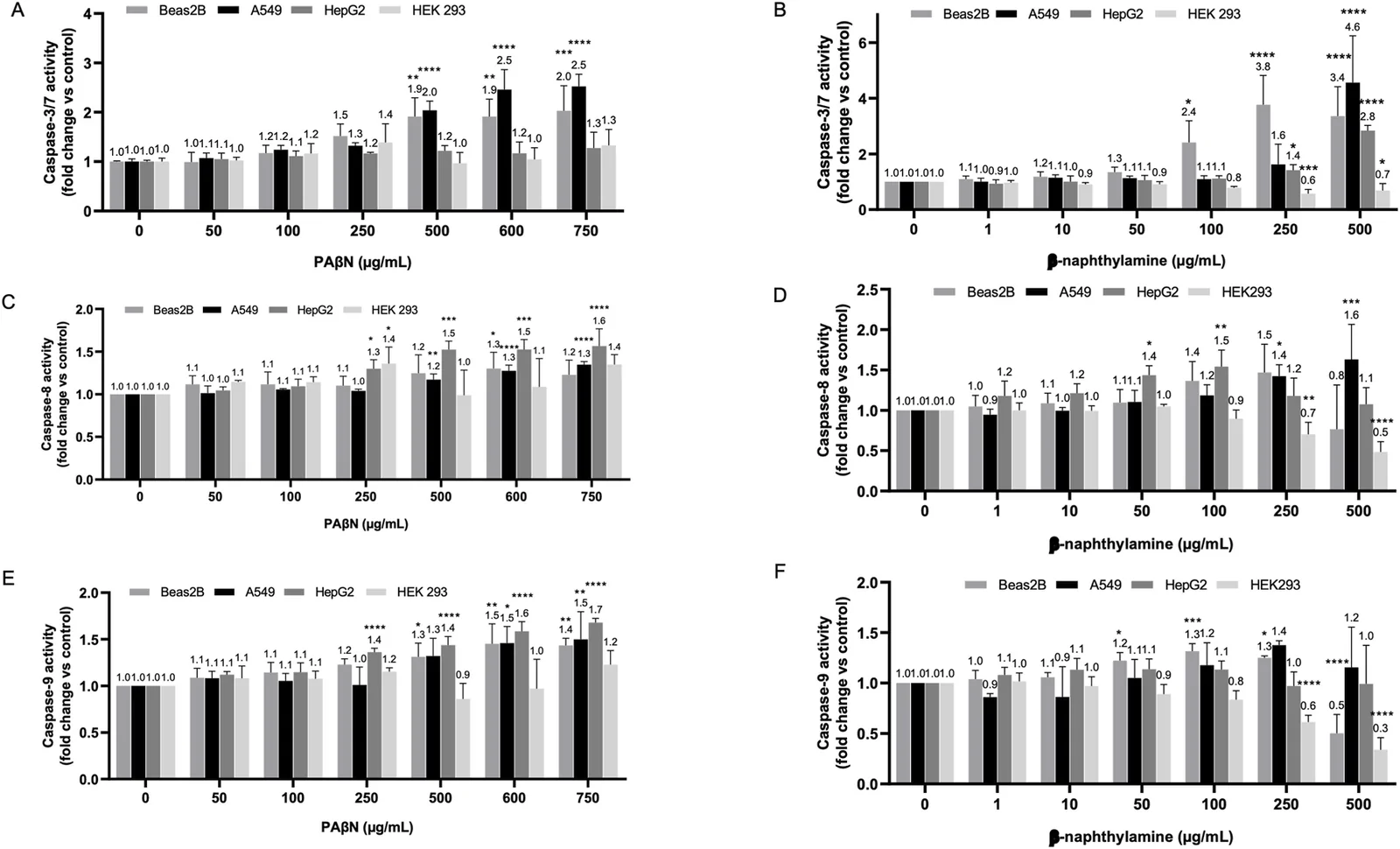
Differential activation of caspases by PAβN and β-naphthylamine in human cell lines. Activities of caspase-3/7 **(A,B)**, caspase-8 **(C,D)**, and caspase-9 **(E,F)** were measured in BEAS-2B, A549, HepG2, and HEK293 cells following 24 h exposure to **(A,C,E)** PAβN (0–1,000 μg/mL; approximately 0–1.9 mM) or **(B,D,F)** β-naphthylamine (0–500 μg/mL; approximately 0–3.5 mM). Data are expressed as fold change relative to untreated control cells and presented as mean ± SD (n = 3). Statistical significance was determined relative to the corresponding untreated control (0 μg/mL): *p < 0.05, **p < 0.01, ***p < 0.001, ****p < 0.0001.

PAβN induced activation of the intrinsic apoptotic pathway in A549 and BEAS-2B cells, as evidenced by significant increases in caspase-9 and caspase-3/7 activities at high concentrations (500–750 μg/mL; 962.5–1,443.8 µM; Figure 6). Activation of caspase-8 was also observed at 600–750 μg/mL, indicating the involvement of the extrinsic apoptotic pathway. In HepG2 cells, PAβN significantly increased caspase-8 and caspase-9 activities from 250 μg/mL (481.3 µM), whereas no significant activation of caspase-3/7 was detected (Figure 6). This dissociation between initiator caspases and caspase-3/7 suggests an incomplete apoptotic response or a blockade downstream of caspase-8 and caspase-9 activation. Despite the induction of ROS production, HEK293 cells did not exhibit significant activation of any of the caspases examined.

In contrast, β-naphthylamine induced activation of caspase-9 from 50 μg/mL (349.2 µM) and caspase-3/7 from 100 μg/mL (698.4 µM) in BEAS-2B cells, supporting the involvement of the intrinsic apoptotic pathway. Activation of caspase-8 was observed in HepG2 cells from 50 μg/mL and in A549 cells only at the highest concentration tested, indicating cell type-dependent activation of the extrinsic pathway. Caspase-3/7 activity increased in HepG2 cells from 250 μg/mL onward. At 500 μg/mL, caspase-3/7 and caspase-9 activities decreased in BEAS-2B and HEK293 cells, coinciding with the marked loss of metabolic activity and increased cytotoxicity observed in the Alamar Blue and LDH assays, respectively (Figures 1, 2).

### 3.3 Description of the interacting regions in the two structures of MexB in complex with PAβN

#### 3.3.1 Structure of MexB-PAβN solved by crystallography

We first solved the structure of MexB in complex with PAβN by crystallography (MexB-PAβN-xtal, PDB ID 33WJ), showing several blobs of density not filled with protein atoms. Note that there are two trimers of MexB in the asymmetric unit of the crystal (P1 space group), but only one trimer contains PAβN (chain B of the 33WJ). They were mostly localized in the membranous domain, at the exception of two extra densities in the porter domain of the T-monomer. As the resolution at which was solved the structure was low (3.56 Å), the forms of these extra densities were not clear enough to easily attribute them. Nevertheless, at the same time, we solved the structure of an uncomplexed form of MexB at 3 Å (PDB ID 33XI) revealing a similar density for only one of the two density blobs present in the T cavity, above the switch-Loop, in the deep binding pocket (DBP). This observation dispelled some of our doubts concerning the presence of a PAβN molecule in our solved structure. The common density was attributed to a molecule of DDM, used as solubilization detergent, even if the density was not big-enough to contain more than the sugar heads of DDM (Supplementary Figure S3).

The second blob is localized in the proximal binding pocket (PBP) close to the entry, and could correspond to a molecule of PAβN. The structure of MexB-PAβN-xtal was also compared with the structures of unassembled MexB already present in the PDB (2V50, 3W9I, 3W9J, 6IIA), the two first structures corresponding to MexB in DDM (Sennhauser et al., 2009), 3W9J to MexB in complex with the non-polymer ligand P9D (Nakashima et al., 2013), a pyridopyrimidine-based inhibitor, and 6IIA to a structure of MexB solved with Lauryl Maltose Neopentyl Glycol (LMNG) as detergent solubilizer (Sakurai et al., 2019). In the two structures solved with DDM, one detergent molecule was present in the T-monomer with a common position for the sugar heads, and the tails oriented toward the entry of the pocket but lining on the two opposite walls of the cavity (Figure 7). This comforted us in the presence of a DDM molecule at this position in our MexB-PAβN-xtal structure. On the contrary, at the position where we built PAβN in the MexB-PAβN-xtal structure, none of the two MexB structures solved with DDM (2V50 (3Å), 3W9I (2.7Å)) presented electronic density, definitely dispelling our doubts on the proper attribution of this density to PAβN. In our MexB crystal structure (PDB ID 33XI), we observe two densities at the same position that are not big enough to accommodate PAβN.

**FIGURE 7:**
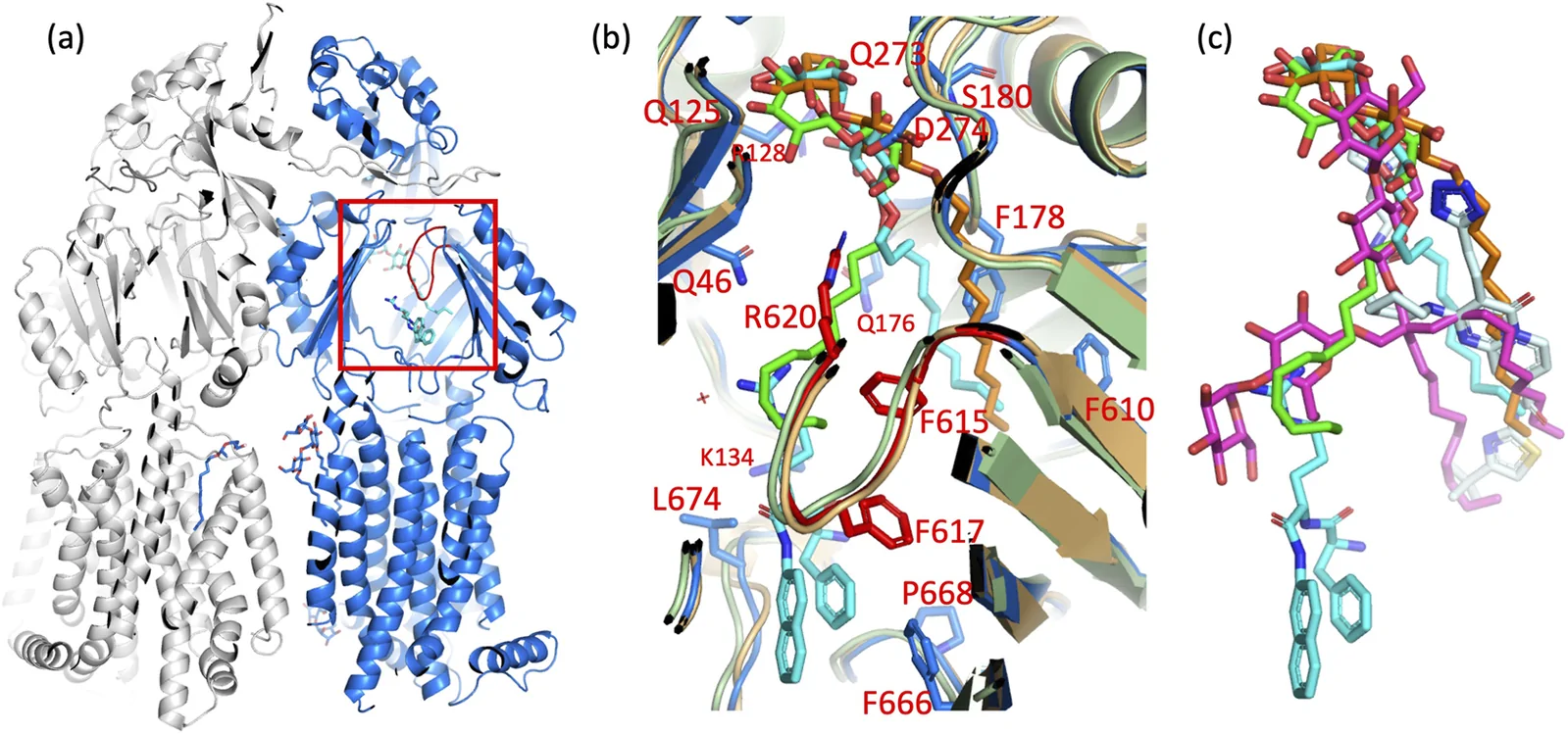
Structure of MexB in complex with PAβN solved by crystallography (MexB-PAβN-xtal). **(a)** Structure of the trimer with the T-monomer in blue and the L- and O- monomers in grey. The switch-Loop is colored in red. The PAβN and the DDM molecule are represented in cyan ball and stick. **(b)** Superposition of the MexB-PAβN-xtal structure with the two structures of MexB solved with DDM as solubilization detergent (2V50 in green and 3W9i in light-orange). The amino acids discussed in the text are from the MexB-PAβN-xtal structure and are shown in ball and stick. **(c)** Superposition of the ligands from the MexB-PAβN-xtal structure (DDM and PAβN) with those present in the other structures of isolated MexB (DDM from 2V50 in green, DDM from 3W9i in orange, LMNG from 6iia in magenta, and P9D from 3W9j in light-blue).

The environment of PAβN is mainly constituted of hydrophobic amino-acids (Phe615, Phe617, Phe666, Pro668, Leu674) comprising those of the switch-Loop, even if the arginine side chain of this tri-peptide is in a more favorable region for a hydrophilic amino-acid (Gln46, Lys134, Arg620). The two aromatics of PAβN are making a stacking together and are parallel to the aromatic cycle of Phe666, even if too far to make a stacking. Phe617 on the contrary is oriented perpendicularly to the aromatic ring of the phenylalanine group of the PAβN. The DDM molecule has been built deeper in the efflux pathway. Its sugar head is surrounded by amino-acids of more hydrophilic properties (Gln46, Gln125, Arg128, Gln273, Asp274, Arg620), where the tail is positioned in a hydrophobic region (Gln176, Phe178, Phe610, Phe615). The switch-Loop separates the two ligands, especially the amino-acids Phe615 and Arg620, common to the two interacting interfaces. When comparing the T-cavity from the other MexB structures solved as unassembled, the different ligands (DDM, LMNG, P9D inhibitor) are all localized in the DBP in close proximity of Phe178, questioning why PAβN is not localized at this position where are usually found the structurally characterized inhibitors. It was hypothesized that this was due to the presence of the molecule of DDM at the expected position, as the structure was obtained by soaking native crystals with a solution of PAβN. Several tries of co-crystallization were performed, each time issuing with one molecule of DDM in the deep binding pocket of MexB. This suggested DDM is a good substrate of MexB and that the molecule was already present from the early steps of purification.

#### 3.3.2 Structure of MexB-PAβN solved by cryo-EM

To get rid of DDM, it has been decided to solve the structure of the MexB/PAβN complex by cryo-EM after reconstitution in lipidic nanodiscs. The preparation of MexB insertion in the nanodiscs requires the use of biobeads that suck the detergent, forcing the protein to surround itself with lipids, but sucking also all the molecules of detergent present inside the cavities, releasing the DBP. The structure of MexB in complex with PAβN was solved by cryo-EM at 2.31 Å resolution and will be called MexB-PAβN-cryo. In this structure, the resolution is not homogenous (Supplementary Figure S4). The membranous domains are solved at low resolution, with the Loose monomer presenting zones in several helices with almost no density (Supplementary Figure S4b). Nevertheless, the amino acids involved in the proton transfer (D407, D408, K939) are properly deffined and adopt the classically described orientation whatever the LTO conformation (Eicher et al., 2014). The pourly defined regions correspond to the helices 2, Iα, 7, 11, 12 located on the surface of the monomer L, and to the random coil ^G^869-871^Q^ of the hoisting-loop region, required for the large conformational changes of the subdomains (Zwama et al., 2017). At the contrary the periplasmic domains are perfectly defined, even for the Loose monomer, making possible to add 366 water molecules to the structure (Supplementary Figure S4c). It has not been possible to obtain a density at a better resolution for the Loose domain in spite of a tremendous effort to optimize its density by testing different treatment approaches listed below: the use of different mask, - the number of initial 2D classes and 3D classes considered, - being very drastic on the number of conserved particles, which was possible thanks to the large number of particles (1.5 million particles) firstly extracted from the 10,483 collected movies. This poor resolution can be due to the presence of the nanodisc that lets the protein diffuse freely in the lipids, or to an intrinsic property of the monomer L to be more flexible in its membranous domain.

In the periplasmic domain, one extra density was identified in the T-monomer in which was positioned a PAβN molecule (Supplementary Figure S4d). The resolution of the protein in this region is very good, but the density of the PAβN is not perfect. It is easy to see that there are three branches in the density that could correspond to the three branches of the PAβN molecules, but it is not sufficiently defined to be absolutely sure of their nature, highlighting the EPI is mobile in the cavity. Nevertheless, it has been positioned in the most probable position (Supplementary Figure S4d) which is also coherent with the environment (Figure 8). The naphthylamide aromatics are stacked between Phe615 from the switch-Loop and F178. The phenylalanine group is located in a region filled with aromatic amino acids (Phe617, F628, F136), and the arginine group is in a slightly less hydrophobic environment with Y327 and M630.

**FIGURE 8:**
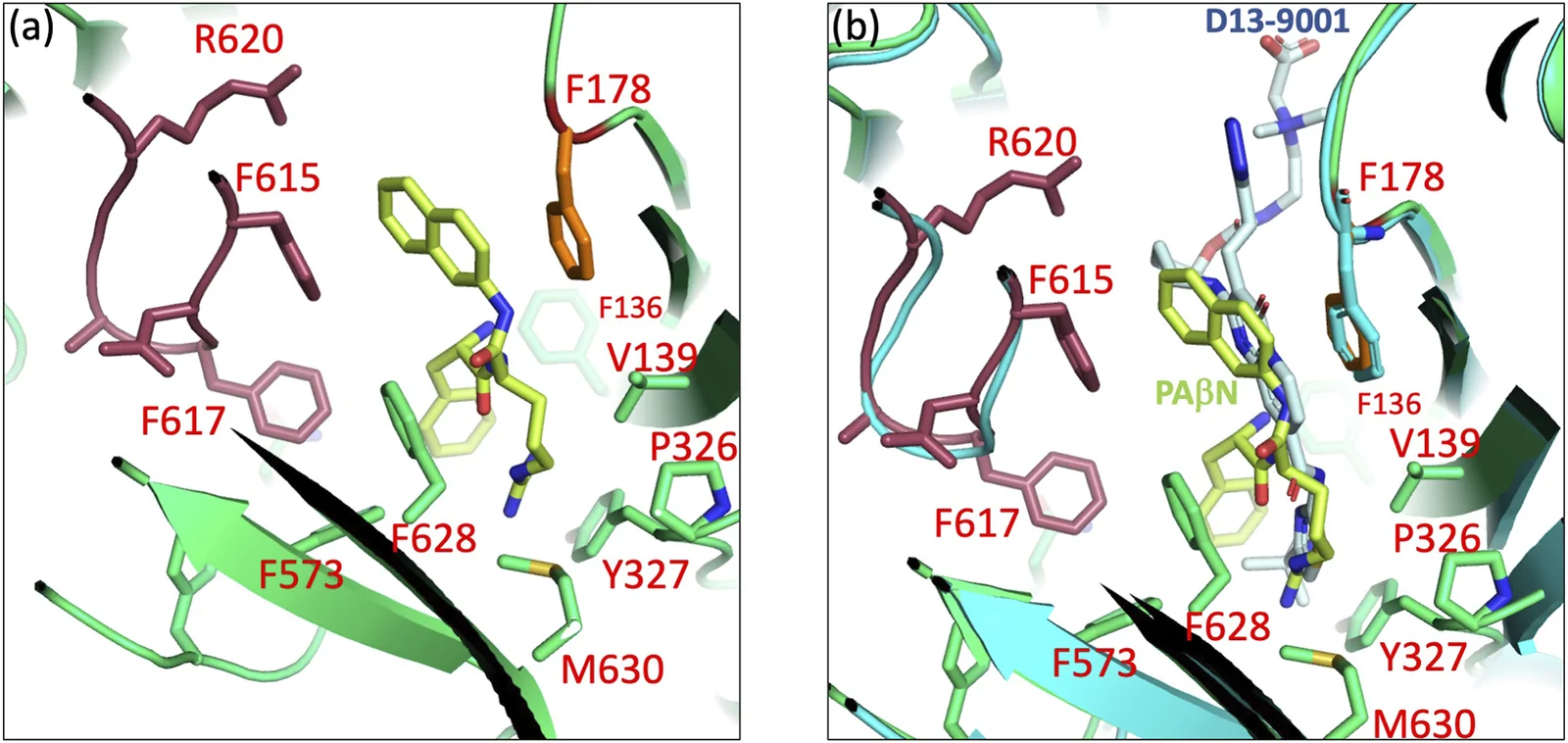
**(a)** Environment of the PAβN in the structure of MexB-PAβN-cryo. All the amino-acids located in close proximity of the EPI are drawn in sticks. The switch-Loop is colored in red, the F178 is in orange, the protein is in green and the PAβN is in lime. **(b)** Same figure on which has been superposed the structure of MexB (cyan) in complex with the EPI D13-9001 (light-blue) (3w9j).

When comparing the position of PAβN with the one of the pyridopyrimidine derivative D13-9001 EPI (Yoshida et al., 2007) solved with MexB (3W9J; (Nakashima et al., 2013)) (Figure 8b), it can be seen that the pyridopyrimidine and the piperidin cycle are positioned where is the naphthylamide of the PAβN, and the thiazol is aligned with the arginine group, which validates the interacting region of the PAβN EPI and so its correct positioning.

#### 3.3.3 Comparison of the two structures of MexB-PAβN

A superposition of the MexB-PAβN-xtal (33WJ) and the MexB-PAβN-cryo (32VC) structures has been performed, highlighting the two different positions of the PAβN in the T-monomer (Figure 9a). In the MexB-PAβN-xtal structure, a molecule of DDM is present where is find the PAβN in the MexB-PAβN-cryo structure (Figure 7), avoiding the PAβN to enter in the DBP. In the MexB-PAβN-cryo structure there is no density at the place where is positioned the EPI in the MexB-PAβN-xtal structure (Figure 9b). Most of the amino-acids present in the environment of PAβN-cryo are superposed with the exception of F178 which is turned to accommodate the DDM molecule. At the contrary, the presence of PAβN-xtal in the PBP entails some modifications in the binding region (Figure 9b), especially on the region which corresponds to the (T676)-loop described in AcrB (Athar et al., 2023).

**FIGURE 9:**
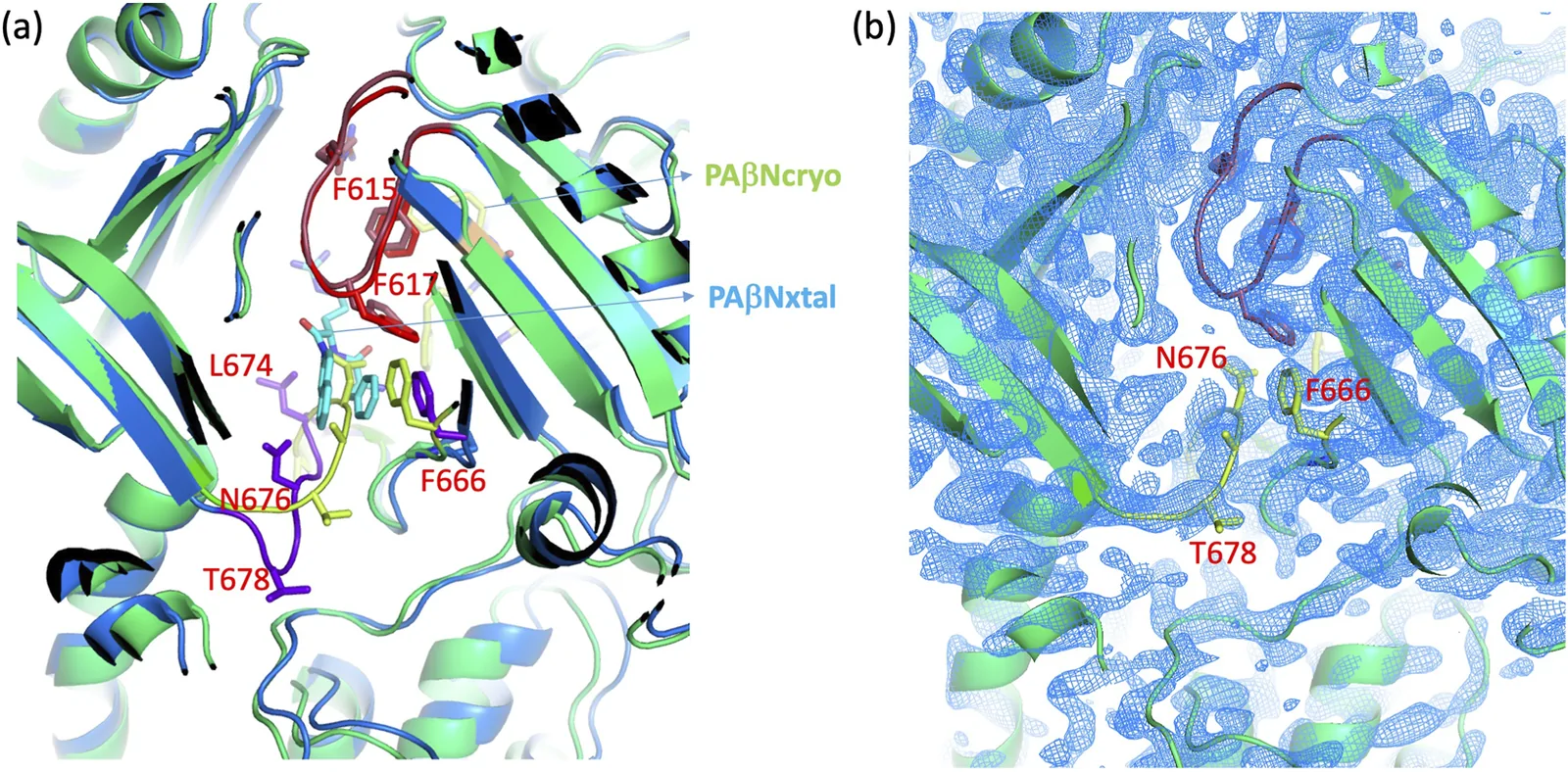
Comparison of the two structures of MexB in complex with PAβN, focusing on the MexB-PAβN-xtal PAβN binding pocket in the PBP. **(a)** Superposition of the two structures highlighting the movement of the E673-T678 and F666-P668 loops. **(b)** Electronic density of the MexB-PAβN-cryo structure, highlighting the absence of PAβN in the structure on this region.

In the MexB-PAβN-xtal structure, the region E673-T678 is pushed towards the membrane domain in order to accommodate the presence of the PAβN. Asn676-cryo is located at the position of PAβN-xtal on the phenylalanine group. In a lower extend, Phe666-Pro668 is also pushed away to further enlarge the cavity. The comparison of these two structures highlight the high adaptivity of the cavities in the T-monomer, certainly thanks to the presence of a lot of loops in the two binding pockets, as described in the analysis of the meta-stable states of MexB (Simsir et al., 2021).

## 4 Discussion

### 4.1 How to use PAβN as a control for RND efflux pumps drug development

#### 4.1.1 Selection of PAβN concentrations for microbiological assays

PAβN is one of the most widely used reference compounds for the phenotypic evaluation of RND efflux pump inhibitors. Because it has been extensively used across numerous bacterial strains and in combination with a wide variety of antibiotic classes, there is a large literature documenting its activity and efficacy (Lomovskaya et al., 2001; Coban et al., 2004; Mesaros et al., 2007). In addition to its RND efflux pumps inhibition role, it also presents bacterial envelop permeabilization effects when used at higher concentration as previously described (Lamers et al., 2013).

The concentrations of PAβN used in this study as adjuvant to the three tested antibiotics align with the range commonly reported for standard antibiotic potentiation assays. It presents an additive effect at 50 μg/mL whatever the tested β-lactam, and decreases the MIC by a fourfold to an eightfold factor depending on the tested antibiotic at 100 μg/mL. At this concentration, there is still no cell membranous envelop perturbation effect as proved by the constant MIC value obtained when tested on the PA14-ΔAB strain. At 200 μg/mL (385 µM), a membranous envelop perturbation effect of PAβN is observed, and a marked growth inhibition when used at higher concentration. Even if not tested in the same conditions, this effect is in line with previous reports describing outer membrane-permeabilizing properties of PAβN. Lomovskaya et al. (2001) showed that no significant outer membrane permeability was observed on PAO1wt from 0 to 128 μg/mL, only on PAO1-ΔMexA was observed an increase of outer membrane permeability at 70 μg/mL. As the human pathogenic Gram-negative bacteria all share similar outer membrane composition, it can be expected that the concentration at which PAβN will show a permeabilization effect will be similar for most of them. Nevertheless, it has been shown that *P. aeruginosa* is less permeable than *E. coli* (Yoshimura and Nikaido, 1982), so it is recommended to use strains deleted of the RND genes coding for the studied efflux pump to verify the integrity of the cellular envelop at the used PAβN concentration. At lower concentrations, when using PAβN as a reference compound for the development of new EPIs, it is recommended to test two to three different concentrations, here 50 and 100 μg/mL, as its effect is not equivalent on all the RND pumps from all the Gram-negative bacteria. As PAβN is more a competitive substrate than an EPI, its target specificity has to be taken into account. This aspect has not been totally explored, but PAβN has also been shown to modulate MexXY-OprM-mediated resistance, with effects that depend on the antibiotic substrate (Mesaros et al., 2007; Charkhi, 2020). These differences may be due to a different repartition of the charges in the transporter cavity (Gregor et al., 2025). Nevertheless, our study confirms the frontier between the two faces of PAβN effects to be in between 100 and 200 μg/mL.

#### 4.1.2 Toxicity

##### 4.1.2.1 Differential cellular effects of PaβN and β-naphthylamine

PAβN is a dipeptide derivative containing a β-naphthylamide moiety, and hydrolysis of the corresponding amide bond could, in principle, result in the release of β-naphthylamine, even if this has not been investigated under our experimental conditions. The present study provides a comparative assessment of the cellular responses induced by PAβN and β-naphthylamine in four human cell lines representing different cellular contexts. Although both compounds induced oxidative stress, mitochondrial alterations and apoptotic signalling, their cellular toxicity profiles were clearly distinct. PAβN is widely used as a reference efflux pump inhibitor (Lomovskaya et al., 2001) and has also been reported to exert cytotoxic effects (Lomovskaya and Bostian, 2006; Fleeman et al., 2018). Our results show that PAβN induced relatively limited effects on overall cell viability and metabolic activity, despite measurable cellular stress at higher concentrations, whereas β-naphthylamine induced substantially stronger cytotoxic responses. This difference remained evident after conversion to molar concentrations, indicating that it cannot be explained solely by differences in molecular weight.

A notable feature of PAβN exposure was the dissociation between metabolic activity and plasma membrane integrity. While Alamar Blue reduction remained largely preserved, LDH release increased at higher concentrations, indicating that alterations in membrane integrity can occur without an equivalent loss of metabolic activity. This is consistent with the heterogeneous effects previously reported for PAβN in mammalian cell models and supports a strong dependence on cell type, exposure conditions and endpoint assessed (Fleeman et al., 2018; Machado et al., 2017; Kaur et al., 2021).

Differences in physicochemical properties may contribute to the greater biological activity of β-naphthylamine. Although the two compounds display comparable predicted lipophilicity, β-naphthylamine is substantially smaller and has a markedly lower topological polar surface area than PAβN (Supplementary Table S3), which may favour membrane permeation and intracellular accumulation. However, cellular uptake was not directly assessed, and this interpretation remains speculative. The stronger activity of β-naphthylamine is also consistent with its established toxicological profile, including bladder toxicity and carcinogenicity following metabolic activation (Centre international de recherche sur le cancer, 2012; Czubacka and Czerczak, 2020), as well as previously reported cytotoxicity, oxidative stress and DNA damage in engineered CHO cells (Habil et al., 2022).

The different kinetics observed for PAβN and β-naphthylamine also raise the possibility that transformation of PAβN could contribute to some of its cellular effects. Because PAβN contains a β-naphthylamide moiety, hydrolysis could, in principle, release β-naphthylamine. However, PAβN stability and β-naphthylamine formation were not assessed under the present experimental conditions, and this possibility therefore remains hypothetical. LC-MS analysis of PAβN stability and transformation products would be required to determine whether such a mechanism occurs.

Overall, PAβN cannot be considered biologically inert toward mammalian cells, but its effects were generally observed at relatively high concentrations and were strongly cell-type and endpoint dependent. In contrast, β-naphthylamine produced stronger and more rapid cellular injury.

##### 4.1.2.2 Cell-type-specific relationships between oxidative stress, mitochondrial dysfunction and cytotoxicity

The integrated analysis of ROS production, mitochondrial membrane potential (Δψm), ATP levels and cytotoxicity revealed distinct response patterns for PAβN and β-naphthylamine (Table 2). Importantly, oxidative and mitochondrial alterations did not consistently translate into loss of viability or activation of apoptotic pathways, indicating that cellular stress responses were not necessarily predictive of overt cytotoxicity.

**TABLE 2:** Comparative summary of the cellular responses induced by PAβN and β-naphthylamine (β-Naph) in BEAS-2B, A549, HepG2, and HEK293 cells**.**

Endpoint	BEAS-2B	A549	HepG2	HEK293
Cell viability	PAβN: ↔ β-Naph: ↓↓↓	PAβN: ↔ β-Naph: ↓↓	PAβN: ↔ β-Naph: ↓↓	PAβN: ↔ β-Naph: ↓↓↓
LDH realase – Membrane damage	PAβN: ↑ (high concentrations) β-Naph: ↑↑	PAβN: ↑ (high concentrations) β-Naph: ↑↑	PAβN: ↔/↑ (high concentrations) β-Naph: ↑↑↑	PAβN: ↑ (high concentrations) β-Naph: ↑↑↑
ROS production – Kinetics	PAβN: Delayed ↑ β-Naph: Early ↑↑	PAβN: Delayed ↑ β-Naph: Early ↑↑	PAβN: Early ↑↑↑ and persistent β-Naph: Early ↑↑↑	PAβN: Delayed ↑↑ β-Naph: Early ↑↑↑
Mitochondrial membrane potential (Δψm)	PAβN: Hyperpolarization → Depolarization β-Naph: ↔	PAβN: Hyperpolarization → Depolarization β-Naph: ↔	PAβN: Depolarization β-Naph: Depolarization	PAβN: Hyperpolarization → Depolarization β-Naph: ↔
ATP content	PAβN: ↓ β-Naph: ↓	PAβN: ↓ β-Naph: ↓	PAβN: ↔ β-Naph: ↔	PAβN: ↓ β-Naph: ↑
Caspase activation	PAβN: ↑↑↑ β-Naph: ↑↑	PAβN: ↑↑↑ β-Naph: ↑	PAβN: ↑↑↑ β-Naph: ↑↑↑	PAβN: ↔ β-Naph: ↔

The table summarizes the effects of both compounds on cell viability, membrane damage, ROS, production, mitochondrial membrane potential (Δψm), ATP, content, and caspase activation. For PAβN, most effects were predominantly observed at the highest concentrations tested, although the concentration dependence varied between endpoints and cell lines. Arrows indicate the direction and relative magnitude of the response (↑, increase; ↓, decrease; ↔, no marked change), with additional arrows indicating greater response intensity. “Early” and “Delayed” describe the kinetics of ROS, production, while “persistent” indicates sustained ROS, production over the assessed time course. “Hyperpolarization → depolarization” denotes a biphasic change in Δψm.

The response to PAβN in A549 and BEAS-2B cells was consistent with a progressive transition from cellular stress toward mitochondrial and cellular dysfunction at higher concentrations. The transient increase in Δψm at intermediate concentrations may reflect an adaptive response to increasing cellular stress, followed by loss of mitochondrial homeostasis at higher concentrations (Zorova et al., 2018). At these concentrations, mitochondrial alterations were accompanied by ATP depletion and increased LDH release, with apoptotic signalling particularly evident in BEAS-2B cells. β-naphthylamine induced a more rapid response, with early oxidative stress followed by mitochondrial alterations and cellular injury.

In contrast, HEK293 and HepG2 cells illustrated that oxidative and mitochondrial stress did not necessarily progress toward classical apoptotic or cytotoxic responses. In HEK293 cells, PAβN induced delayed ROS production together with mitochondrial and energetic alterations at high concentrations, but without significant activation of the caspases investigated. HepG2 cells provided an even clearer example of this dissociation: PAβN induced marked and sustained ROS production while ATP levels, viability and membrane integrity remained comparatively preserved over much of the concentration range, with more pronounced mitochondrial and cellular alterations occurring only at the highest concentrations. β-naphthylamine again produced a more coordinated response, characterized by early ROS production followed by mitochondrial depolarization and increased LDH release.

These findings therefore indicate that ROS production alone was insufficient to predict the extent of cellular injury. Although oxidative stress can contribute to mitochondrial dysfunction and apoptotic signalling (Redza-Dutordoir and Averill-Bates, 2016), the present results show that these events were not consistently coupled across cell lines. In particular, sustained oxidative stress could occur in the absence of marked ATP depletion or loss of viability, suggesting that cells may initially compensate for oxidative and mitochondrial disturbances before reaching a threshold associated with overt injury. Conversely, when mitochondrial and energetic alterations became more pronounced, as observed at higher concentrations, they were more frequently accompanied by membrane damage and, in some cell lines, apoptotic signaling. Thus, the progression from oxidative stress to cellular injury appears to depend strongly on cellular context and on the ability of each cell type to maintain mitochondrial and energetic homeostasis.

##### 4.1.2.3 Implications for the use of PAβN as an efflux pump inhibitor

These findings have implications for the use of PAβN as an efflux pump inhibitor. Most cellular effects observed here occurred at relatively high concentrations, generally approaching or exceeding the millimolar range, whereas PAβN is commonly used at substantially lower concentrations in bacterial efflux inhibition studies, as are the antibiotics and existing adjuvants in therapeutic. The present results indicate that PAβN should not be considered biologically inert toward mammalian cells, particularly at high concentrations or following prolonged exposure.

These results emphasize the importance of considering concentration, exposure duration and cellular context when interpreting experiments using PAβN. This is particularly relevant when comparing bacterial and mammalian systems, for which the effective exposure conditions may differ substantially. Overall, the present study shows that PAβN and β-naphthylamine induce distinct, concentration- and cell-type-dependent cellular response profiles, and that oxidative stress, mitochondrial alterations and cytotoxicity do not necessarily develop in parallel. The conceptual model presented in Figure 10 summarizes these response patterns and highlights the importance of considering compound-specific effects and cellular context when interpreting the biological effects of PAβN. This also highlights the importance to study the stability of EPIs under proteolysis conditions to verify resulting fragments will not be responsible for toxicity when used in therapeutic conditions.

**FIGURE 10:**
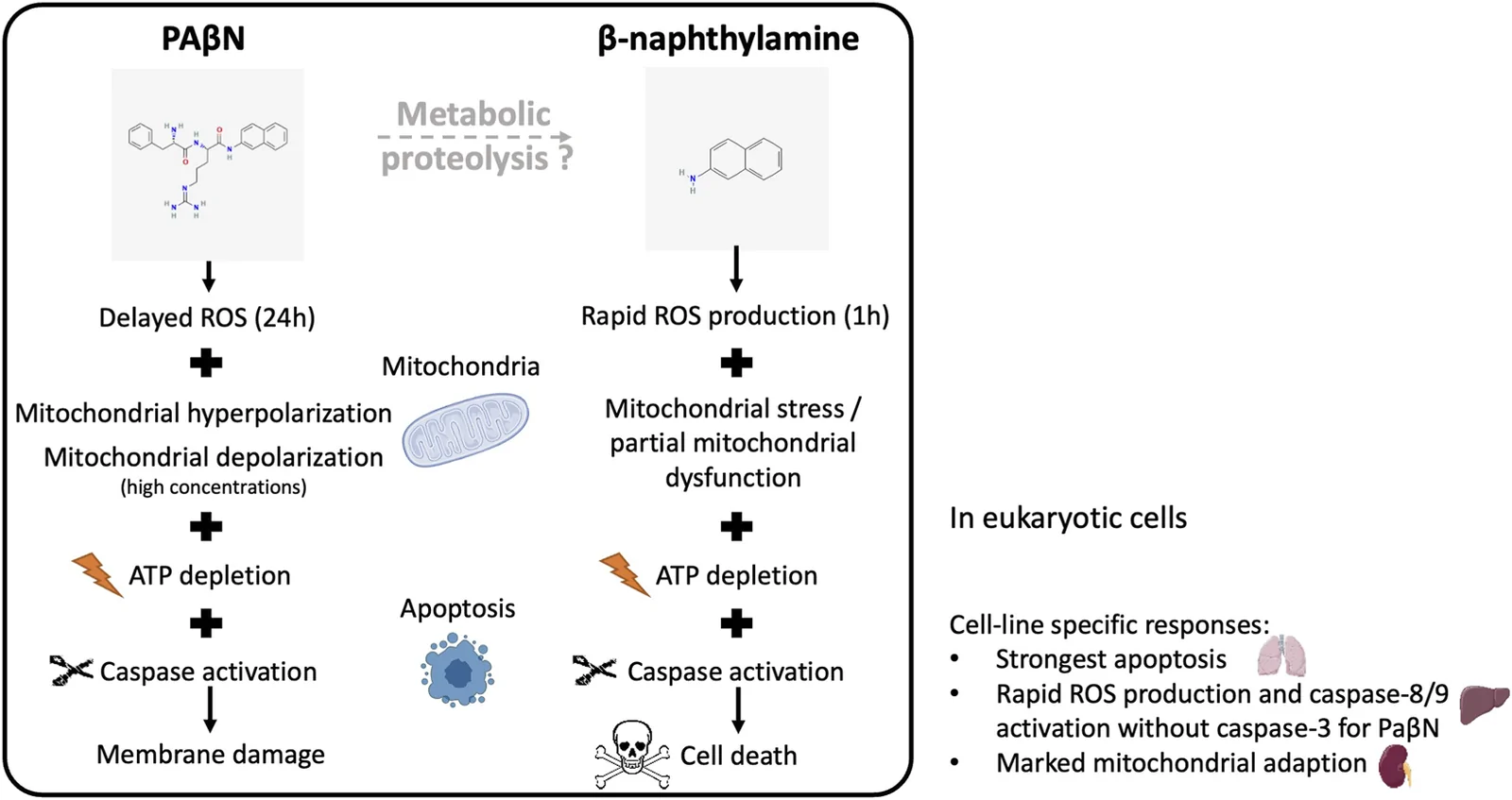
Proposed model summarizing the eukaryotic cellular responses induced by PAβN and β-naphthylamine. The model integrates the experimental observations obtained *in vitro* following exposure to concentrations substantially higher than those expected under physiological conditions. Solid arrows represent responses supported by experimental data. Dashed arrows indicate hypothetical relationships that were not directly investigated in the present study. The figure is intended as a conceptual framework summarizing the observed cellular responses rather than a definitive mechanistic pathway.

### 4.2 Comprehension of the efflux pathway used by PAβN in RND transporters

Even before the first structure of the AcrB transporter from *E. coli* has been solved in 2002 (Murakami et al., 2002), a tremendous effort has been made to develop EPIs, as the blockade of RND efflux pumps was already envisaged as a good strategy to restore the use of antibiotics. Nevertheless, it has not been possible to describe a mechanism for the efflux through RND transporters before solving the structure of AcrB as an asymmetric trimer (Seeger et al., 2006). These structures (PDB code 2GIF and 2DHH) revealed the alternative conformations taken by the protein, later called L, T, O, to transport its substrates through a complex pathway (Alav et al., 2021). Since that time, a lot of different molecules have been synthesized to attain this goal (Compagne et al., 2023), most of them being tested on bacterial strains using PAβN as a control, but only one structure has been solved so far of a RND transporter in complex with this EPI (Yu et al., 2005). This structure (1T9Y), solved at 3.64 Å resolution, corresponds to AcrB from *E. coli* solved in a crystallographic space group in which the trimer is reconstituted by the crystallographic axis, avoiding the conformation modifications required for substrate efflux. In this AcrB/PAβN structure, the molecule of PAβN is present in the PBP at the entry of the cavity, which could indicate this AcrB monomer corresponds to the Loose conformation. Nevertheless, when calculating a Root Mean Square Deviation (RMSD) between this monomer and the three monomers from each here solved structures of MexB, it appears to fit better with the Tight conformation whatever the structure with a RMSD of 1.45Å for MexB-PAβN-cryo and 1.53Å for MexB-PAβN-xtal, calculated respectively on 865 and 882 Cα. Most of the differences correspond to movements inside the PC2 domain forming the left wall of the cavity entry (see (Athar et al., 2023) for the nomenclature). This AcrB monomer seems to adopt an intermediate conformation, with the entry of the cavity slightly more closed than in a L-monomer, coherent with the entry of a molecule in the cavity, but without enlargement of the DBP, certainly blocked by the imposed crystallographic symmetry. To go further in this analysis, the structure of AcrB-PAβN was superposed to the T-monomers of the structures solved in this study, highlighting the different positions taken by PAβN during its transport (Figure 11).

**FIGURE 11:**
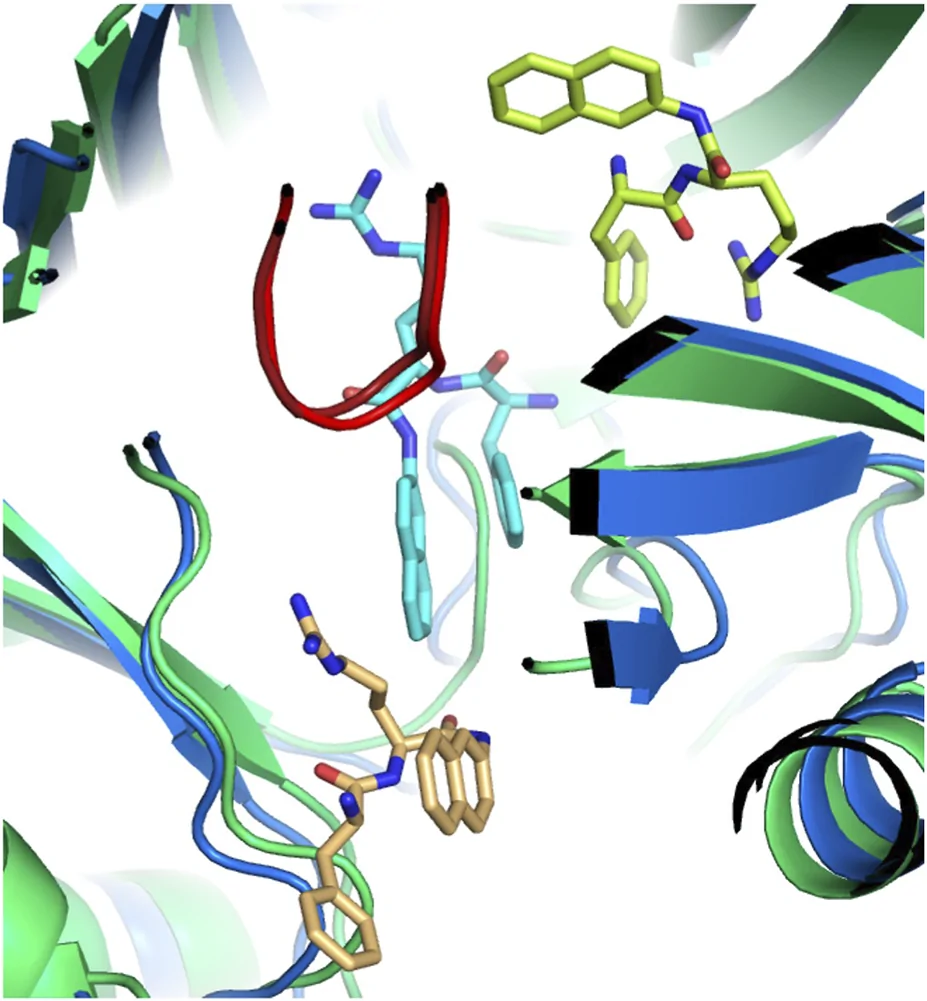
Superposition of the two structures solved in this study with the PAβN molecule from the AcrB structure solved by crystallography in a space group where the trimer was formed via one crystallographic three-axis. PAβN-cryo, PAβN-xtal and PAβN-AcrB are colored respectively in lime, in cyan, and in light orange. The protein structure of AcrB is not shown for clarity.

As already said, PAβN-AcrB is located at the entry of channel 2, at the beginning of the PBP, PAβN-cryo is in the DBP like most of the substrates that have been solved in complex with RND transporters so far, and PAβN-xtal is in an intermediate position, in between the PBP and the DBP before the switch-Loop. These three very different positions could reveal the efflux pathways of one molecule taken in charge from the periplasm, that would first follow the left edge of the cavity, and stay there below the switch-Loop, waiting for the DBP to be free (here filled with a DDM molecule in the MexB-PAβN-xtal structure). When the path is released, it will then reach the right wall of the cavity, in the DBP on the other side of the switch-Loop. It can be noticed that the conformation of the PAβN molecule is very different in the three structures, the three side chains not making contact in the AcrB structure, the naphthylamide being stacked on the phenyl in the MexB- PAβN-xtal structure, where the phenyl is parallel to the guanidinium of the arginine in the MexB-cryo structure. These very different orientations of the three pieces of the PAβN molecule reflect its high flexibility even when entered into the efflux tunnel, even if at the low resolution of AcrB-PAβN (3.64Å) and of MexB-PAβN-xtal (3.56Å), it is difficult to discuss of their precise positions. Nevertheless, the global forms of the different densities in the three structures clearly indicate a different orientation of the three fragments forming the PAβN, leaving no doubt that PaβN is not effluxed in a rigid conformation. This could indicate that there is no real binding of the molecule with the protein before reaching the DBP, but some resting zones, reflecting its suggested role as a competitive substrate more than an inhibitor, at least when referring to the admitted definition. This is in accordance with the *in silico* study performed on the binding of PAβN in the cavities of AcrB (Vargiu and Nikaido, 2012), showing very large differences between the initial docking of the molecule and the final position calculated by molecular dynamic. This position corresponds to none of the ones observed in the three structures, but to another intermediate close to the switch-Loop. Nevertheless, in this MD simulation, it was indicated the implication of residues from the PBP (G675), the interface (F617, E673), and the cleft (F664, F666, L668) in the interaction with inhibitors. They correspond to some of the residues from the two loops E673-T678 and F666-P668 highly modified when comparing MexB-PAβN-xtal and MexB-PAβN-cryo, precising the loops involved in the transport of the molecules through the PBP-DBP cavities. For the MexB-PAβN-cryo structure, PAβN is located in the DBP and is surrounded by amino-acids classically described to be involved in the interaction with EPIs (Gervasoni et al., 2023), a resting position before the opening of the path for final extrusion (Athar et al., 2023).

Concerning the lower resolution of the membrane domain, this has already been observed when solving the structure of MexB reconstituted in nanodiscs (Glavier et al., 2020), and can be due to the free diffusion of the protein in the lipids of the nanodisc. The very poor resolution of half of the membranous domain of the L conformer is more puzzling. This observed flexibility encompasses the membranous region forming the bottom of the channel 1 entry in the L monomer, entry used for taking in charge the small or lipophilic substrates diffusing in the inner membrane (Zwama et al., 2018; Athar et al., 2023). The upper part of helix 8, the random coil ^G^869-871^Q^, that belongs to channel 1 is also poorly define. It corresponds to a hoisting-loop that has been described to be important for the conformational changes of the subdomains (Zwama et al., 2017), adopting a coil structure only in the Loose monomer and being helical in the two others, and it has been suggested to be involved in the energy coupling mechanism. This function could explain why channel 1 and part of the membranous domain is less resolved, as it could be advantageous to have a floppy structure to accommodate more easily different kinds of ligands.

## 5 Conclusion

In order to develop new drugs that could be used as adjuvants to restore the efficiency of the current antibiotic pharmacopeia, the development of efflux pump inhibitors (EPIs) is an interesting strategy. Nevertheless, most of the molecules that present interesting effects on Gram-negative bacteria are not pushed further due to high toxicity or poor specificity. This underlines the need to tackle the problem by different approaches in parallel, and to be able to compare results with a well-established control molecule, like PAβN that is used as EPI reference in most of the studies dealing with RND efflux pumps.

For drug-design development of new EPIs targeting the transporter MexB, structures of MexB in complex with PAβN have been solved, bringing interesting insights into the pathway taken by substrates and inhibitors to reach the DBP. The experimental conditions were verified for the use of PAβN on Gram-negative bacteria, confirming the range at which this molecule can be used without cellular envelope perturbation. On the contrary, the results obtained in toxicology revealed a lack of toxicity of PAβN at concentrations corresponding to classical therapeutic use of antibiotics and adjuvants in clinic. This suggests that the observed toxicity attributed to PAβN could be due to *in vivo* metabolism proteolysis of PAβN, this hypothesis being sustained by the toxicity of β-naphtylamine mimicking the β-naphtylamide fragment of PAβN. By this multiapproach study, it has been possible to complete the current knowledge on PAβN, precising the conditions of use of this unavoidable control for the development of new specific EPIs.

## Data Availability

The datasets generated for this study can be found in the protein data bank (PDB) at https://www.rcsb.org/ with the code 33XI for MexB and 32VC and 33WJ for the cryo‐EM and crystallographic structures of MexB/PAbN respectively.
