# Supplementary material for: Revisit of the admitted control RND efflux pumps’ inhibitor PAβN: use limitations, and structural revelation in complex with MexB
Source: Front Pharmacol. 2026 Oct 19;17:1906963. doi: 10.3389/fphar.2026.1906963 (PMC13725085; doi:10.3389/fphar.2026.1906963)
Supplement: Supplementary file 1 [file Supplementaryfile1.docx]

Supplementary Material

# 1 Supplementary Tables

**Supplementary Table 1: MexB-PAβN-xtal data statistics**

|  | **MexB-PaβN complex** | **MexB** |
| --- | --- | --- |
| **PDB ID** | 33WJ | 33XI |
| Data collection |  |  |
| **Wavelength** (Å) | 0.9677 | 0.9677 |
| **Resolution range** (Å) | 45.81 - 3.56 (3.69 - 3.56) | 47.78 - 3.0 (3.11 - 3.00) |
| **Space group** | P 1 | P 1 |
| **Unit cell**  *a*, *b*, *c* (Å)  *α*, *β*, *γ* (°) | 125.93 135.71 153.20  86.19 69.11 87.56 | 125.01 134.34 151.50  87.18 69.53 88.14 |
| **Total reflections** | 401 588 (41 677) | 650 818 (69 668) |
| **Unique reflections** | 106 421 (9 391) | 173 168 (17 439) |
| **Multiplicity** | 3.8 (3.8) | 3.8 (3.9) |
| **Completeness (%)** | 92.54 (82.70) | 92.76 (94.55) |
| **Mean I/sigma(I)** | 6.79 (1.03) | 5.68 (0.66) |
| **Wilson B-factor** | 112.41 | 91.68 |
| **R-merge** | 0.1524 (1.259) | 0.1482 (1.752) |
| **R-meas** | 0.1781 (1.462) | 0.174 (2.031) |
| **R-pim** | 0.09161 (0.742) | 0.09027 (1.025) |
| **CC1/2** | 0.953 (0.606) | 0.978 (0.416) |
| **CC*** | 0.988 (0.869) | 0.994 (0.766) |
| Refinement |  |  |
| **Reflections used in refinement** | 104827 (9340) | 171287 (17438) |
| **Reflections used for R-free** | 1099 (98) | 1800 (183) |
| **R-work** | 0.2854 (0.4738) | 0.2845 (0.4340) |
| **R-free** | 0.2975 (0.4296) | 0.3020 (0.3977) |
| **CC(work)** | 0.840 (0.610) | 0.857 (0.432) |
| **CC(free)** | 0.849 (0.741) | 0.883 (0.392) |
| **Number of non-hydrogen atoms** | 47031 | 46998 |
| **macromolecules** | 46640 | 46640 |
| **ligands** | 391 | 358 |
| **Protein residues** | 6147 | 6147 |
| **RMS(bonds)** | 0.007 | 0.011 |
| **RMS(angles)** | 1.10 | 1.44 |
| **Ramachandran favored (%)** | 95.25 | 95.43 |
| **Ramachandran allowed (%)** | 3.74 | 3.54 |
| **Ramachandran outliers (%)** | 1.01 | 1.03 |
| **Rotamer outliers (%)** | 8.25 | 9.70 |
| **Clashscore** | 19.79 | 23.23 |
| **Average B-factor** | 127.08 | 101.87 |
| macromolecules | 127.12 | 101.99 |
| ligands | 122.73 | 85.98 |

Statistics for the highest-resolution shell are shown in parentheses.

**Supplementary table 2 Conversion of tested concentrations from mass concentration (µg/mL) to molar concentration (µM)**

| concentration (µg/mL) | PaβN (µM) | β-naphthylamine (µM) |
| --- | --- | --- |
| 0.1 | - | 0.70 |
| 1 | - | 6.98 |
| 10 | - | 69.8 |
| 50 | 96.3 | 349.2 |
| 100 | 192.5 | 698.4 |
| 250 | 481.3 | 1746.0 |
| 500 | 962.5 | 3492.6 |
| 600 | 1155.0 | - |
| 750 | 1443.8 | 5238.9 |
| 1000 | 1925.0 | 6985.1 |

PAβN MW = 519,47 g/mol; β-naphthylamine MW = 143.19 g/mol

Concentrations are presented in µg/mL throughout the figures of the manuscript because treatments were prepared and administered on a mass basis. Equivalent molar concentrations are provided here to facilitate comparison between compounds with different molecular weights.

**Supplementary Table 3 Selected physicochemical properties of PAβN and β-naphthylamine.**

| Property | PaβN (dihydrochloride)* | β-naphthylamine |
| --- | --- | --- |
| Molecular formula | C_25_H_3_0N_6_O_2_ | C_10_H_9_N |
| Molecular weight used for concentration conversion (g/mol) | 519.47 | 143.19 |
| Molecular weight of neutral structure (g/mol) | 446.54 | 143.19 |
| Consensus LogP (SwissADME) | 2.02 | 2.39 |
| TPSA (Å²) | 148.62 | 26.02 |
| Rotatable bonds | 12 | 0 |
| H-bond donors | 5 | 1 |
| H-bond acceptors | 4 | 0 |
| Predicted GI absorption | Low | High |
| Predicted BBB permeation | No | Yes |
| Predicted P-gp substrate | Yes | No |

*PAβN was used as the dihydrochloride salt in the present study. Molecular descriptors were calculated from the neutral structure using SwissADME.

Physicochemical descriptors were obtained using SwissADME and are provided to facilitate interpretation of differences in cellular responses observed between PAβN and β-naphthylamine.

# 2 Supplementary Figures

**Supplementary Figure 1.** Effect of PAβN on propidium iodide uptake and cell number in human cell lines. A549/BEAS-2B (A and D), HepG2 (B and E), and HEK293 (C and F) cells were treated for 24 h with PAβN (0–1000 µg/mL; ~0–1925.0 µM). Propidium iodide uptake is shown as fold change vs control, and cell number is expressed relative to control (mean ± SD, n = 3). *p < 0.05, **p < 0.01, ****p < 0.0001 vs control.

**Supplementary Figure 2.** Time-dependent ROS production induced by PaβN. Intracellular ROS levels were measured in BEAS-2B, A549, HEK293, and HepG2 cells using the H_2_DCFH-DA assay. (A–D)  Cells were exposed to PaβN for 3 h and 6 h. Data are expressed as fold change relative to untreated control cells and presented as mean ± SD (n = 3). Statistical significance: * compared with the corresponding untreated control (0 µg/mL) (* p < 0.05, ** p < 0.01, *** p < 0.001, **** p < 0.0001).

**Supplementary Figure 3**. Electron density map of the T monomer of MexB- PAβN crystal structure. MexB is represented in blue cartoon with side chains in stick, PAβN is represented in cyan and DDM is represented in yellow. Map contour is 1 sigma.

**Supplementary Figure 4**. Quality of the structure of MexB-PAβN solved by cryo-EM. a) MexB map colored in function of the resolution. b) MexB-PAβN-Cryo-EM map drawn at 3 sigma. c) Example of the Cryo-EM density quality around the switch-Loop (red) of the T-monomer, making possible to resolve the water molecules. d) Electronic density at 3 sigma centered on the PAβN colored in lime.
